# Recurrent and system-specific model-attribution patterns in PISA 2022 science performance across nine education systems

**DOI:** 10.1371/journal.pone.0351534

**Published:** 2026-09-18

**Authors:** Meng Liu, Xin Bai, Xiaohong Li, Wei Jin, Weifeng Jin

**Affiliations:** 1 School of Artificial Intelligence in Traditional Chinese Medicine, Zhejiang Chinese Medical University, Hangzhou, China; 2 School of Medical Technology, Zhejiang Chinese Medical University, Hangzhou, China; 3 School of Pharmaceutical Sciences, Zhejiang Chinese Medical University, Hangzhou, China; Nanjing University, CHINA

## Abstract

Using science data from the 2022 Programme for International Student Assessment (PISA), we examined survey-weighted descriptive association and model-attribution patterns across a fixed, non-probability cohort of nine education systems (78,685 students). Because PISA science plausible values are drawn from a latent population distribution generated with a conditioning model that uses background information, the analysis describes survey-weighted descriptive associations rather than independent individual predictions. Seven machine-learning pipelines were evaluated descriptively across 20 repeated school-grouped held-out partitions in each system. The genetic algorithm-optimized backpropagation neural network (GA-BPNN) was retained as the focal architecture for Shapley additive explanations (SHAP), without claiming systematic or universal superiority. Alternative training-only median/mode imputation produced system- and metric-specific performance changes. The four sensitivity-supported variables—ESCS, HOMEPOS, MATHEFF, and ST255Q01JA—formed a conservative subset of the eight variables that recurred under the primary top-35 screening procedure. Feature-screening stability was assessed under the high-missingness quartile stress test, whereas held-out performance and SHAP magnitude/rank variation were assessed only under the full alternative median/mode imputation workflow; the sample-restriction analysis was not used to assess changes in SHAP ranks. A cross-architecture check showed substantial but incomplete agreement between GA-BPNN SHAP and XGBoost TreeSHAP. These results describe recurrent and system-specific attribution patterns across the nine education systems. They do not support causal inference, individual diagnosis, or generalization to all PISA systems; missing-data analyses did not resolve missing-not-at-random (MNAR) mechanisms or residual selection bias.

## 1. Introduction

The Programme for International Student Assessment (PISA) is administered every three years to evaluate 15-year-old students’ academic achievement in reading, mathematics, and science through a standardized assessment system [1,2]. Beyond test scores, it systematically collects individual data on students’ socioeconomic backgrounds, learning attitudes, and language environments, as well as school-level information on resource allocation and instructional organization [1,3]. Overall, this program provides a data foundation for cross-nationally comparable monitoring of educational performance and for describing variables associated with achievement patterns in large samples [1,4,5].

Recent PISA studies have applied machine-learning models to achievement data and used SHAP to summarize how fitted models allocate importance to student, family, and school variables [6–13]. This framing requires care because PISA plausible values are multiple imputations intended for population-level inference rather than observed individual scores [2,14]. In addition, the latent-trait conditioning model uses background and questionnaire information that can overlap with candidate predictors. A model fitted to plausible values may therefore recover associations already represented in the PISA population model rather than predict an independent measure of individual achievement. The present study consequently treats machine learning as a descriptive tool for mapping survey-weighted descriptive association and model-attribution patterns across education systems [5,15].

Using PISA 2022 science data, this study analyzes a fixed, non-probability analytical case cohort of nine Level 3 education systems with heterogeneous questionnaire completeness [1,2]. It addresses two questions: how stable are the descriptive model-fit patterns across repeated school-grouped holdouts within these named cases, and which model-attribution patterns recur across systems or remain system-specific? Seven pipelines were compared: a standard backpropagation neural network (BPNN), genetic algorithm-optimized BPNN (GA-BPNN), particle swarm optimization-optimized BPNN (PSO-BPNN), simulated annealing-optimized BPNN (SA-BPNN), Extreme Gradient Boosting (XGBoost), random forest (RF), and support vector regression (SVR). GA-BPNN was retained as the focal model for SHAP analysis, while the held-out model summaries provide descriptive context rather than a formal test of systematic superiority. The study does not validate individual PISA scores or an external outcome, and all conclusions are limited to survey-weighted descriptive association patterns within the nine named cases [2,14,16,17].

## 2. Materials and methods

### 2.1. Data description

#### (1) Sample composition.

Scientific literacy enables citizens to make informed decisions [4]. This study uses data from the OECD PISA 2022 assessment. Although PISA 2022 covered 81 participating education systems and economies, the public student questionnaire database contains no valid sample for Cyprus; therefore, the initial dataset includes 613,744 15-year-old students from the remaining 80 participating systems.

Educational systems were classified into four tiers based on weighted mean scientific literacy scores: Level 1 (<410), Level 2 (410–484), Level 3 (484–559), and Level 4 (≥559). Among them, 25 systems were Level 1, 25 Level 2, 29 Level 3, and 1 Level 4.

Questionnaire completeness differed substantially across PISA systems. We calculated missingness using the raw PISA 2022 student file and the frozen union of 114 base variables entering the final model-ready feature sets. Across Level 3 systems, mean variable missingness ranged from 0.158 to 0.463. The nine analyzed systems had a mean of 0.274, compared with 0.319 in the other 20 Level 3 systems. These summaries document the cohort but do not imply that missingness was random or that the nine systems were selected by an optimized missingness threshold. Missingness diagnostics by performance level and inclusion status are reported in S1 Appendix Table S6.

The analytical cohort was a fixed, non-probability set identified by the codes AUS, BEL, CZE, FIN, GBR, IRL, KOR, LTU, and POL. The historical selection did not use a reconstructible system-level missingness cutoff or another explicit inclusion rule. Membership is transparent through the exact system list, the 488-variable candidate eligibility registry used for screening, the frozen 114-variable union of model-ready feature sets, and the complete Level 3 missingness table in S1 Appendix Table S10; these materials do not convert the historical choice into a reproducible sampling rule. The final sample includes 78,685 students, and Table 1 reports full-sample weighted means and standard errors based on all ten plausible values and the PISA replicate weights. Findings are limited to these nine named cases and are not generalized to all Level 3 systems or to the full PISA population.

**Table 1:** Full-sample descriptive information for the nine education systems.

Education system	Weighted Mean	Standard Error	Sample Size
Australia	507.00	1.93	13437
Belgium	490.58	2.48	8286
Czech Republic	497.74	2.3	8460
Finland	510.96	2.5	10239
Ireland	503.85	2.26	5569
Korea	527.82	3.58	6454
Lithuania	484.46	2.33	7257
Poland	499.16	2.55	6011
United Kingdom	499.67	2.38	12972

Note: Means and standard errors combine the ten science-plausible values and the 80 Fay-adjusted balanced repeated replication (BRR) weights supplied by PISA.

#### (2) Data preprocessing and feature selection.

This study used a nested, train-isolated preprocessing and feature-selection procedure for each education system. PISA uses a multistage sampling design in which schools are sampled before students. Students within the same school may share instructional conditions, resources, and other school-level contextual features. A student-level random split could therefore place students from the same school in both development and test data and produce overly optimistic performance estimates because of within-school dependence. We therefore used the PISA school identifier (CNTSCHID) as the grouping variable and assigned all students from a given school to the same partition. This design evaluates the full analytical pipeline on students from schools not used for model development within the same education system. The 60% missingness threshold was applied at the variable level within the training data of each retained education system and was not used as a direct system-level inclusion criterion. Missing values, including systematic codes (95, 97, 99) and invalid responses, were cleaned and standardized. For model development, the outer training data were further divided into inner training and validation sets using the same school-grouped principle.

Within each development stage, all data-dependent operations were estimated exclusively from the corresponding training partition. Variables exceeding 60% missingness were identified from the training data and removed. Five-neighbor distance-weighted k-nearest-neighbor (KNN) imputation was then performed using the corresponding training data as the exclusive donor pool. Information from the validation or test partition was not used to fit the imputation procedure or modify the training data. Variables coded as discrete response categories were returned to discrete values after imputation. Complete-case feasibility across the system-specific top-35 screened variables is reported in S1 Appendix Table S8.

Weighted Spearman correlations were calculated only from the imputed training data, using the final student weight and the 10 science plausible values. Correlations across the plausible values were aggregated for variable ranking, and the false discovery rate (FDR) was controlled at 1% using the Benjamini-Hochberg procedure [18]. The 35 eligible variables with the highest absolute weighted correlations were retained for each education system. Only after selection of these 35 base variables were categorical recoding, one-hot encoding, and Z-score standardization fitted using the training data and applied to the corresponding validation or test data using the training-fitted transformations.

After the model-development settings had been fixed using the inner training and validation data, the complete preprocessing and feature-selection sequence was refitted using the full outer training data. Within each outer split, the held-out test data were used only for final evaluation and did not contribute to missingness filtering, KNN fitting, variable ranking or selection, categorical encoding, standardization, model-setting decisions, or stopping decisions.

The top-35 threshold was used as a pragmatic cap to keep model dimensionality comparable across education systems while limiting expansion after one-hot encoding. It was not treated as a theoretically optimal threshold or selected by comparing outer-test model performance. To assess threshold sensitivity, the weighted Spearman screening audit was repeated at top-25, top-35, and top-45 retention thresholds. A variable was considered recurrent in an education system only when it was selected in all 20 school-grouped outer splits for that system. A complementary least absolute shrinkage and selection operator (LASSO) and elastic net sensitivity check was also conducted to assess whether the recurrent predictor structure was solely an artifact of univariate Spearman screening. The threshold-sensitivity audit is reported in S1 Appendix Table S9. The LASSO and Elastic Net sensitivity check is reported in S1 Appendix (Supplementary Methods S3).

Two distinct missing-data sensitivity programs were conducted in the same nine systems and 20 school-grouped outer-training partitions per system. The first program was restricted to feature screening. Primary KNN screening was compared with a fresh training-derived median/mode screen; within each outer-training partition, the quartile with the greatest pre-imputation predictor missingness was also removed from model development before the fresh median/mode screen was repeated. The paired held-out partition was unchanged. These screening analyses did not re-estimate focal GA-BPNN held-out performance or SHAP after either the imputation change or quartile exclusion. The second program was a full alternative-imputation analysis that repeated the complete training-only workflow with median imputation for continuous variables and mode imputation for categorical variables in place of five-neighbor distance-weighted KNN. This analysis directly re-estimated the focal GA-BPNN held-out performance and SHAP under the alternative imputation. Neither program establishes MAR, identifies an MNAR mechanism, or eliminates residual selection bias. Complementary missingness, threshold, and cohort diagnostics are reported in S1 Appendix (Appendices A-E).

### 2.2. Treatment of plausible values and complex survey design

#### (1) Accounting for measurement uncertainty using plausible values.

PISA science achievement is a latent population construct represented by ten plausible values, PV1SCIE to PV10SCIE, rather than a directly observed individual score [1,14]. Model fitting and evaluation were repeated separately for each plausible value, and metric estimates were combined across the ten draws. This procedure propagates plausible-value variation into descriptive summaries, but it does not transform plausible values into individual diagnostic scores. The resulting metrics are interpreted as survey-weighted descriptive model summaries within each education system. Because the PISA conditioning model incorporates background information related to some candidate predictors, fitted associations may partly reconstruct information represented in the latent-outcome model and are not interpreted as prediction of an independent achievement measure.

#### (2) Accounting for sampling weights.

The final student weight, W_FSTUWT, was used in full-sample descriptive summaries, model fitting where supported, and aggregate model-fit metrics. Within repeated holdouts, weighting preserves the relative contribution of sampled students in each partition, but it does not by itself convert a partial holdout into a new design-based population estimate [1,16,17].

#### (3) Estimating sampling variance using replicate weights.

The 80 Fay-adjusted balanced repeated replication (BRR) weights were used with all ten plausible values to estimate sampling uncertainty for the full-sample descriptive science means in Table 1 [16,17]. We do not claim design-consistent BRR standard errors for metrics calculated inside partial train/test subsets, because the replicate weights were constructed for the full PISA sample design rather than for the random holdout partitions. Uncertainty in the repeated model evaluation is therefore described by variation across the 20 school-grouped outer splits. Full-sample and partition-based weighting details are reported in S1 Appendix (Supplementary Methods S1).

### 2.3. Model comparison and evaluation framework

The seven models were used as survey-weighted descriptive association-mapping tools rather than as individual diagnostic instruments or stand-alone educational theories. All pipelines were evaluated under the same education-system-specific train-validation-test protocol. For each system, 20 repeated school-grouped held-out partitions assigned approximately 80% of school clusters to training and 20% to a held-out partition. These analyses assess the stability of fitted relationships when applied to students from other sampled schools within the same system; they do not validate an independent achievement outcome or yield new design-representative PISA samples.

To reduce data-leakage risk, the workflow within each outer split was ordered as follows: (1) Students were split into training, internal-validation, and test subsets; (2) system-specific missingness filtering was estimated using training data; (3) KNN imputation was fitted on the training subset and then applied to validation and test subsets; (4) weighted Spearman screening was computed using the training subset only; (5) categorical encoding and standardization were fitted on training data and applied unchanged to validation and test data; (6) model selection and optimization-based initialization used the training/internal-validation subsets; and (7) the test set was held out until final evaluation. The leakage-control protocol is detailed in S1 Appendix (Supplementary Methods S2).

Model fit in the repeated school-grouped held-out partitions was summarized using W_FSTUWT-weighted R², mean absolute error (MAE), mean squared error (MSE), and root mean squared error (RMSE). Each pipeline was fitted and evaluated separately for the ten science plausible values, after which the ten metric estimates were combined to obtain a split-level descriptive model summary. Held-out R² was summarized as mean (SD) across the 20 repeated school-grouped outer splits; complete MAE, MSE, and RMSE summaries are reported in S1 Appendix Table S12. The SD describes split-to-split variation. No design-consistent BRR inference or formal test of model superiority is claimed for these partial holdouts.

The detailed GA, PSO, and SA update equations, fitness/objective functions, stopping criteria, pseudocode, hidden-layer settings, and optimization budgets are reported in S1 Appendix (Supplementary Methods S4-S8 and Tables S1-S3). We therefore interpret the primary model results as comparisons under the 20 repeated school-grouped outer-split protocol rather than as definitive estimates of performance under all possible random partitions.

GA-BPNN was retained as the focal hybrid neural architecture for attribution only after all 180 split-level outer evaluations had been completed. The resulting held-out summaries were then reviewed once in a study-level descriptive audit for the fixed nine-system cohort: within each system, the seven pipelines were ranked by held-out R² (higher is better) and MAE, MSE, and RMSE (lower is better) across the 20 school-grouped outer splits; each system and metric received equal weight in the aggregate rank sum. GA-BPNN had the lowest aggregate rank sum (41.5/36; mean rank 1.153). This operational rule selected one common architecture for the subsequent attribution stage; it did not retroactively alter any split-specific missingness filtering, imputation, feature screening, preprocessing, model fitting, optimization, or stopping decision, and it is not evidence of statistically significant, systematic, or universal superiority. After this study-level choice, the same GA-BPNN pipeline was used for SHAP in every education system, plausible value, and outer split, without system-, variable-, or result-specific model switching. XGBoost TreeSHAP was added as a complementary architecture-dependence check; exact attribution magnitudes and ranks remain model-conditional.

### 2.4. Benchmark model

Model comparison proceeded in two descriptive steps. The hybrid BPNN variants were first placed alongside standard BPNN to show the performance pattern associated with optimization-based initialization. The neural-network pipelines were then placed alongside RF, XGBoost, and SVR baselines [19–21]. All pipelines used the same system-specific partitions and documented settings. The held-out summaries provide comparative context for the focal model; they are not used to infer systematic superiority across education systems.

### 2.5. SHAP-based model interpretation

SHAP was used to summarize how each input contributed to the output of the fitted GA-BPNN model [13]. These values describe the behavior of the focal model conditional on its training data, correlated predictors, and plausible-value target; they are not estimates of independent or causal effects.

The cross-system analysis used SHAP DeepExplainer for each GA-BPNN model fitted in 20 school-grouped outer splits per education system. For each outer split and plausible value, 256 observations were sampled from the corresponding outer-training partition without replacement, with sampling probabilities proportional to W_FSTUWT; the sampling seed was the outer-split seed plus 314159, and SHAP values were computed for all students in each outer-test set. Signed SHAP values for one-hot columns were summed back to the original PISA variable before absolute values were taken. Within each split, global importance was the W_FSTUWT-weighted mean absolute SHAP value across held-out students. Values were then averaged across plausible values and splits, and education systems received equal weight in the cross-system ranking. Additional SHAP aggregation details are reported in S1 Appendix (Supplementary Methods S9).

Architecture dependence was assessed using XGBoost TreeSHAP under the same primary KNN preprocessing workflow and the same 20 paired school-grouped outer splits per education system. For each paired split, agreement with GA-BPNN SHAP was summarized by the Jaccard overlap of the 10 highest-ranked original variables and the Spearman correlation of feature ranks across the 35 primary screening variables. This comparison was designed as a descriptive attribution-sensitivity check rather than a test of performance superiority. Split-level agreement summaries are reported in S1 Appendix Table S15.

Recombining one-hot columns at the original-variable level reduced fragmentation and aligned the display with the PISA questionnaire constructs. Variables not selected in a split contributed zero to the unconditional cross-split summary. Because predictors can be correlated with one another and with variables used in the PISA conditioning model, SHAP values are reported as model-attribution summaries rather than independent real-world effects.

### 2.6. Hyperparameter configuration

Hyperparameter and optimization settings for all evaluated models were fixed before test-set evaluation and are reported in S1 Appendix Tables S1 and S2. The standard BPNN and hybrid BPNN variants shared a feed-forward architecture with one hidden layer, sigmoid hidden activation, 40% dropout, and one continuous output node. The system-specific hidden-layer sizes reported in S1 Appendix Table S3 were retained in the revised analyses: AUS = 14, BEL = 12, CZE = 12, FIN = 14, GBR = 15, IRL = 10, KOR = 13, LTU = 12, and POL = 10. Final training used weighted mean-squared-error loss, with the final student weight incorporated into the loss calculation. Models were trained using the Adam optimizer, learning rate 0.005, weight decay 0.001, batch size 1,024, and a maximum of 400 epochs. Early stopping with a patience of 35 epochs was used to limit overfitting. GA, PSO, and SA were used only as additional initialization stages for the hybrid variants before the same final backpropagation training procedure.

### 2.7. Statistical method

Full-sample science means and their standard errors were estimated as population-weighted descriptive estimates with the final student weight, all ten plausible values, and the 80 Fay-adjusted BRR replicate weights. For each plausible value, repeated model evaluation produced W_FSTUWT-weighted descriptive model summaries. The resulting split-level estimates were summarized as mean ± SD across 20 repeated school-grouped held-out partitions. BRR-based standard errors were not used for partial-holdout metrics.

Spearman rank correlation was used for training-only screening of ordinal and continuous questionnaire variables, with the Benjamini-Hochberg procedure controlling the false discovery rate [18]. The nine education systems form a purposive cohort rather than a probability sample of systems, so no broader inference is drawn from differences between systems.

Supplementary model-comparison diagnostics are treated as exploratory descriptions of the same fixed cohort and are not used to generalize model rankings to other PISA systems. Residual diagnostics are reported in S1 Appendix (Supplementary Methods S10).

### 2.8. Ethics statement

This study used publicly available, de-identified OECD PISA 2022 data. The authors did not access identifiable personal information or interact with participants. Because the dataset was publicly available and de-identified, no additional institutional ethics approval or informed consent was required for this secondary analysis. The study posed no risks to individuals or individual privacy.

## 3. Results

### 3.1. Sample and variable structure across nine education systems

**Fig 1:**
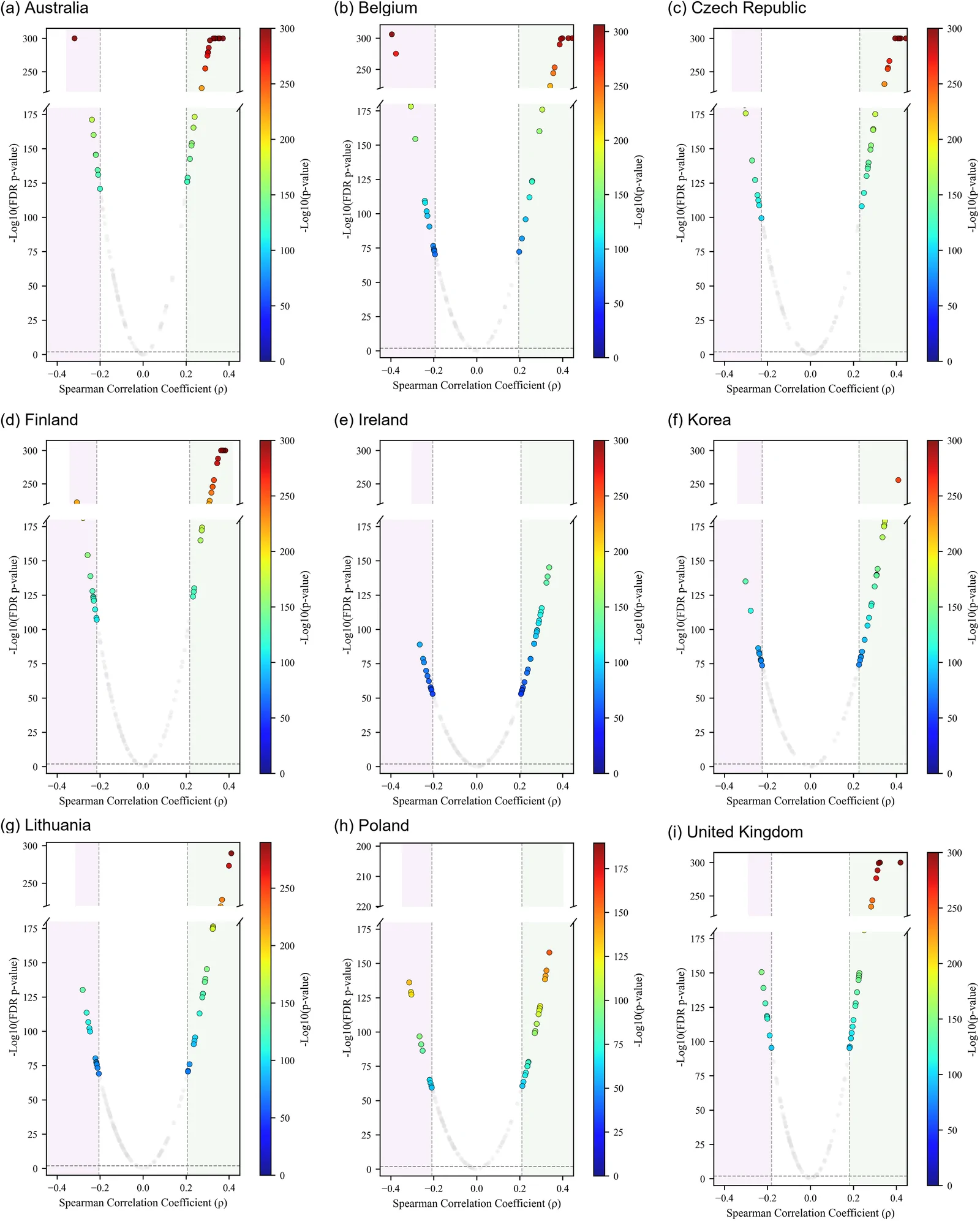
Training-set weighted Spearman screening plots for the nine education systems. The horizontal dashed line marks the FDR-adjusted p-value threshold (q = 0.01), and the vertical dashed guides at |𝜌| = 0.20 are shown for descriptive orientation only. In the primary analysis, the 35 FDR-eligible variables with the largest absolute weighted Spearman correlations were retained in each education system.

The analytical sample included 78,685 students from the fixed cohort comprising AUS, BEL, CZE, FIN, GBR, IRL, KOR, LTU, and POL. These systems all fell within the Level 3 range of mean science scores, but they were not selected by an optimized missingness cutoff. Across the 488-variable candidate eligibility registry used for screening and the frozen 114-variable union of model-ready feature sets, their mean variable missingness was 0.274, compared with 0.319 in the other 20 Level 3 systems; the complete Level 3 table is reported in S1 Appendix Table S10. Table 1 reports full-sample weighted science means, design-based standard errors, and sample sizes. Missingness burden remained associated with lower science performance and the index of economic, social and cultural status (ESCS) in several systems (S1 Appendix Table S7), so residual selection bias cannot be excluded. Training-only weighted Spearman screening is summarized in Fig 1.

**Fig 2:**
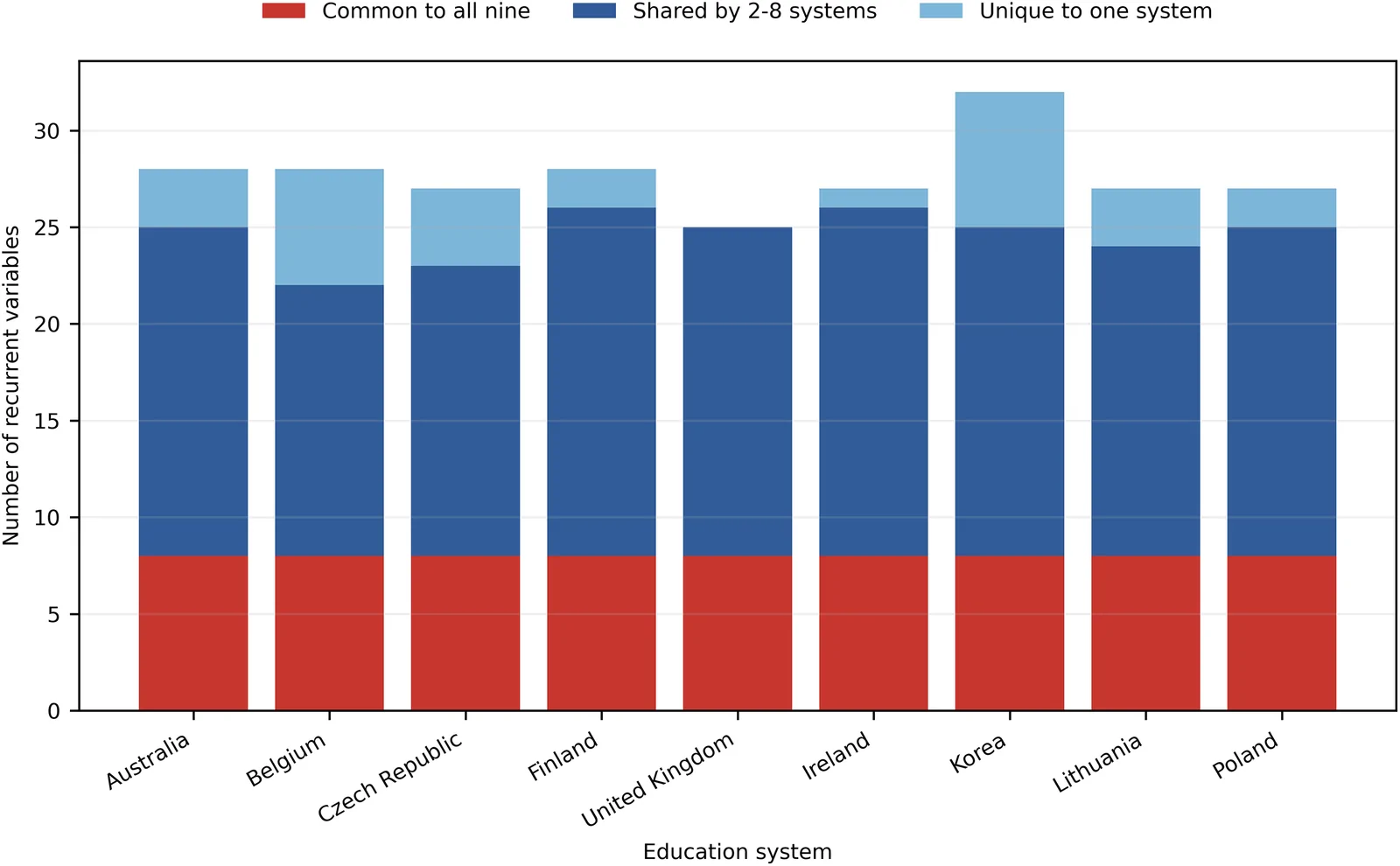
Cross-system feature structure of recurrent top-35 feature sets. Stacked bars show counts in each education system of variables selected in all 20 school-grouped outer splits under the primary top-35 screening procedure, classified as recurrent in nine systems, shared by two to eight systems, or unique to one system.

Fig 2 summarizes the cross-system structure of recurrent top-35 feature sets. For each education system, a variable entered its system-level recurrent set only when it was selected in all 20 school-grouped outer splits under the primary top-35 screening procedure. Eight variables were recurrent in all nine education systems; the remaining variables were shared by two to eight systems or were unique to one system. This structure supports reporting common and system-specific association patterns rather than treating the cohort as a homogeneous population.

### 3.2. Descriptive model-performance patterns

Table 2 summarizes held-out R² across 20 repeated school-grouped outer splits. Complete MAE, MSE, and RMSE results are provided in S1 Appendix Table S12. The split-to-split SDs document partition variability, and these comparisons are descriptive rather than a formal test of model superiority. The common GA-BPNN attribution architecture was selected only after the complete set of split-level held-out summaries had been compiled for the study-level descriptive audit described in Section 2.3. That post-evaluation choice did not affect model development or evaluation within any split, and it does not establish GA-BPNN as a proven best-performing model.

**Table 2:** Descriptive held-out R² summary for seven pipelines across nine education systems.

System	GA-BPNN	PSO-BPNN	SA-BPNN	BPNN	XGBoost	RF	SVR
AUS	0.501 (0.0181)	0.486 (0.0190)	0.485 (0.0184)	0.476 (0.0180)	0.479 (0.0205)	0.444 (0.0198)	0.456 (0.0180)
BEL	0.565 (0.0236)	0.532 (0.0241)	0.543 (0.0245)	0.519 (0.0240)	0.515 (0.0233)	0.543 (0.0260)	0.497 (0.0259)
CZE	0.507 (0.0222)	0.503 (0.0226)	0.486 (0.0224)	0.472 (0.0223)	0.477 (0.0220)	0.453 (0.0249)	0.445 (0.0234)
FIN	0.495 (0.0120)	0.463 (0.0119)	0.473 (0.0118)	0.442 (0.0130)	0.473 (0.0143)	0.434 (0.0143)	0.433 (0.0139)
IRL	0.516 (0.0170)	0.506 (0.0171)	0.513 (0.0179)	0.479 (0.0177)	0.478 (0.0195)	0.461 (0.0189)	0.484 (0.0200)
KOR	0.378 (0.0286)	0.366 (0.0285)	0.344 (0.0300)	0.335 (0.0298)	0.347 (0.0295)	0.325 (0.0330)	0.294 (0.0322)
LTU	0.496 (0.0251)	0.503 (0.0258)	0.484 (0.0256)	0.473 (0.0262)	0.471 (0.0263)	0.449 (0.0282)	0.457 (0.0287)
POL	0.462 (0.0159)	0.446 (0.0171)	0.455 (0.0171)	0.462 (0.0170)	0.463 (0.0184)	0.443 (0.0195)	0.386 (0.0188)
GBR	0.456 (0.0188)	0.422 (0.0204)	0.432 (0.0192)	0.413 (0.0195)	0.409 (0.0227)	0.369 (0.0244)	0.337 (0.0218)

Values are mean (SD) across 20 repeated school-grouped outer splits; estimates for the 10 plausible values were combined within each split. Complete MAE, MSE, and RMSE results are in S1 Appendix Table S12.

Abbreviations: AUS, Australia; BEL, Belgium; CZE, Czech Republic; FIN, Finland; IRL, Ireland; KOR, Korea; LTU, Lithuania; POL, Poland; GBR, United Kingdom.

### 3.3. Recurrent predictors across education systems

Under the primary KNN top-35 screening procedure, eight variables were retained in every outer split of all nine education systems: ESCS, HOMEPOS, MATHEFF, ST255Q01JA, ST290Q03WA, ST290Q05WA, ST290Q07WA, and ST290Q09WA (Table 3). Their recurrence indicates repeated inclusion in this association-mapping workflow, not identical effects or mechanisms. ESCS was retained in all systems by marginal weighted Spearman screening but by both LASSO and Elastic Net in only Australia, Finland, Ireland, Korea, and Lithuania. It was therefore recurrent under Spearman screening but sensitive to regularization.

**Table 3:** Plain-language definitions for the PISA variables analyzed in this study. Definitions are adapted from the OECD PISA 2022 student questionnaire codebook.

Variable	Plain-language definition	Shown in
IC173Q04JA	How often use [digital resources] in lessons in: [Computer science], [information technology], [informatics] or similar lessons.	Fig 3
MATHEFF	Mathematics self-efficacy: formal and applied mathematics – response options reversed in 2022 (WLE)	Fig 3 and Fig 4
ST255Q01JA	How many books are there in your [home]?	Fig 3 and Fig 4
ST294Q05JA	How many days/wk before school: Exercise or practise a sport (e.g., running, cycling, aerobics, soccer, skating, [country-specific])	Fig 3
IC180Q08JA	Agree/disagree: I share made-up information on social networks without flagging its inaccuracy.	Fig 3
ST256Q03JA	How many of these books at [home]: Contemporary literature	Fig 3
IC183Q01JA	Can do with [digital resources]: Search for and find relevant information online	Fig 3
HOMEPOS	Home possessions (WLE)	Fig 3 and Fig 4
ST290Q03WA	How confident in math tasks: Calculating how many square metres of tiles you need to cover a floor	Fig 3 and Fig 4
ST294Q04JA	How many days/wk before school: Work for pay	Fig 3
ST290Q09WA	How confident in math tasks: Solving an equation like 3x + 5 = 17	Fig 3 and Fig 4
ST275Q05WA	How often at school: Solving an equation like 6x² + 5 = 29	Fig 3
HISEI	Highest parental occupational status (ISEI) based on 4-digit human coded ISCO	Fig 3
ST303Q05JA	Agree/disagree: I think there is only one correct position in a disagreement.	Fig 3
ST275Q07WA	How often at school: Solving an equation like 2(x + 3) = (x + 3)(x-3)	Fig 3
ESCS	Index of economic, social and cultural status	Fig 4
ST290Q05WA	How confident in math tasks: Solving an equation like 6x² + 5 = 29	Fig 4
ST290Q07WA	How confident in math tasks: Solving an equation like 2(x + 3) = (x + 3)(x-3)	Fig 4

**Fig 3:**
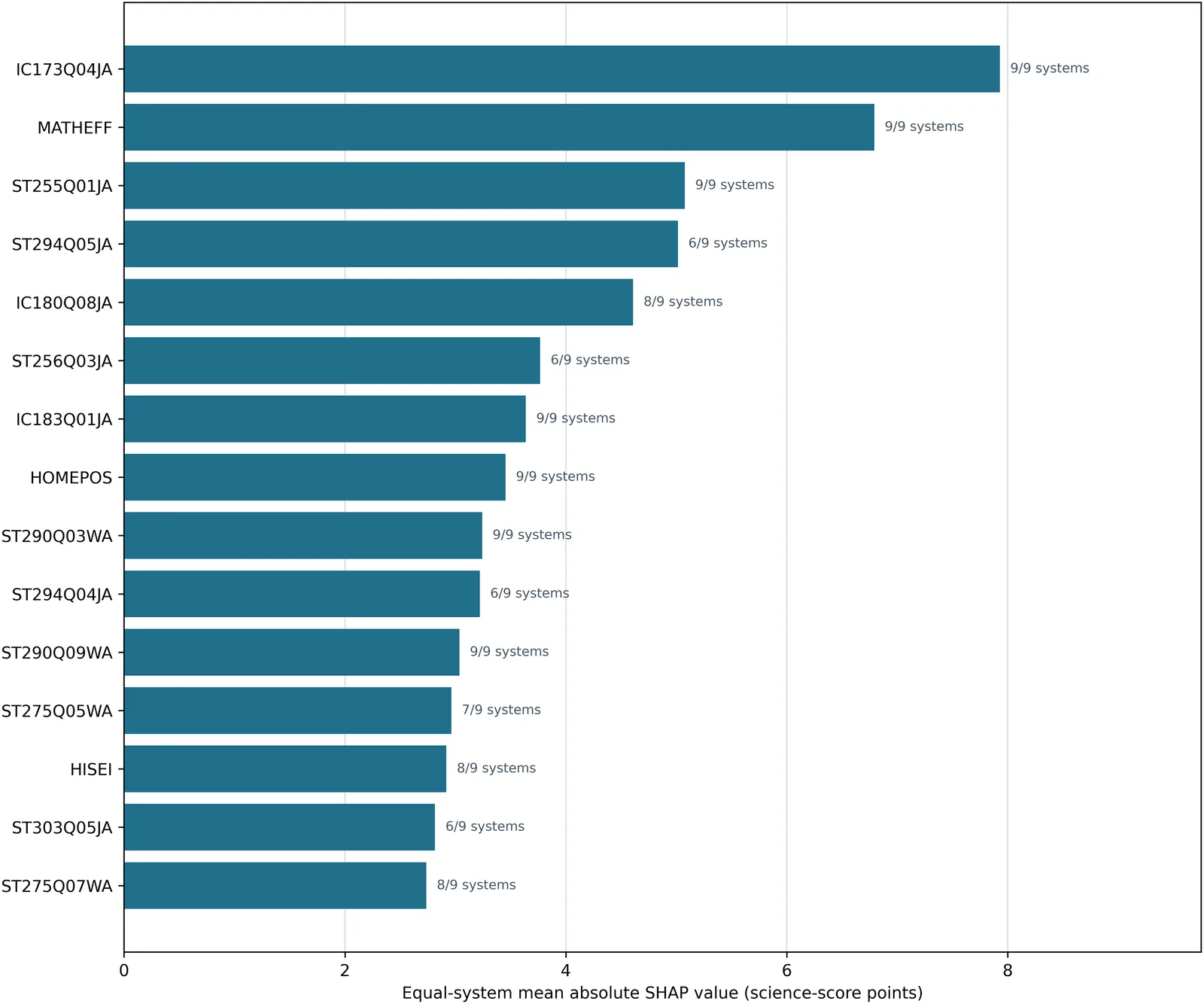
Equal-system global mean absolute SHAP importance for the focal GA-BPNN model. DeepExplainer results are aggregated across 20 school-grouped outer splits and ten plausible-value models per education system after W_FSTUWT-weighted student aggregation and original-variable one-hot collapse.

**Fig 4:**
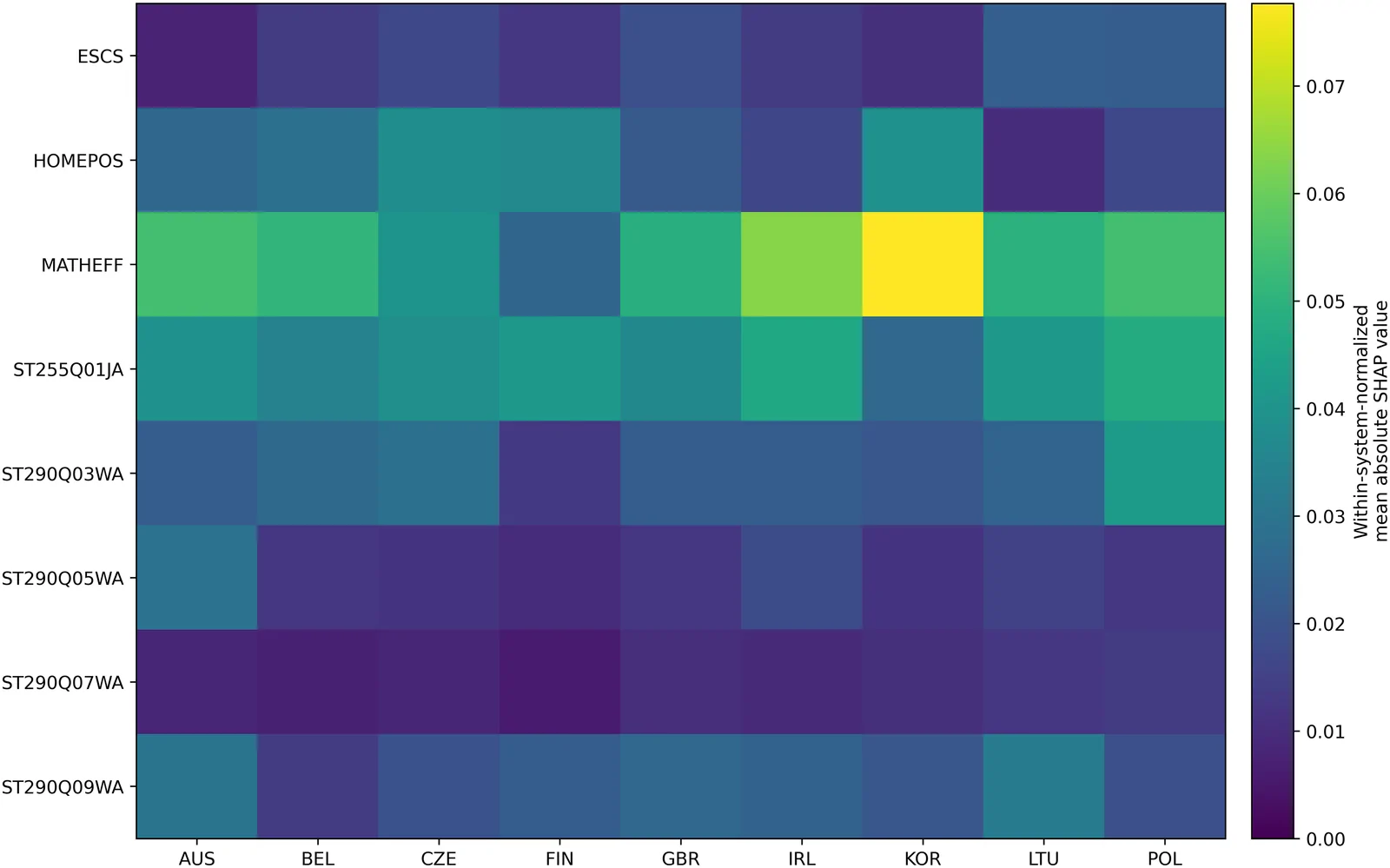
Within-system-normalized mean absolute SHAP importance for eight variables recurrent under the primary KNN top-35 procedure. Each cell summarizes 20 school-grouped outer splits and ten plausible-value models within an education system. The plot is a focal GA-BPNN attribution summary; it does not establish missing-data-invariant feature selection. Plain-language definitions are provided in Table 3.

Threshold sensitivity contextualized this recurrent-variable set. At the stricter top-25 threshold, ESCS, MATHEFF, ST290Q05WA, and ST290Q09WA were recurrent in all nine systems; HOMEPOS was recurrent in seven systems, and ST255Q01JA, ST290Q03WA, and ST290Q07WA were recurrent in eight. All eight primary recurrent variables were recurrent in all nine systems under both top-35 and top-45 retention. At the top-45 threshold, IC173Q04JA and ST290Q01WA were also recurrent in all nine systems. Accordingly, the primary recurrent-variable analysis is described as a top-35 analysis rather than a threshold-invariant set. Counts of education systems in which each variable was recurrent under top-25, top-35, and top-45 screening are reported in S1 Appendix Table S9.

### 3.4. Missing-data sensitivity analysis

As a screening-only check, primary KNN screening was compared with fresh training-derived median/mode screening across the 180 paired outer-training partitions. The mean top-35 Jaccard overlap was 0.504 (range 0.346–0.750) and the mean full-ranking Spearman correlation was 0.783. For variables common to paired top-35 sets, association directions agreed in every comparison. ESCS, HOMEPOS, MATHEFF, and ST255Q01JA remained selected in every split of all nine systems under both procedures. These results concern feature-screening stability only and do not assess held-out GA-BPNN performance or SHAP sensitivity. The paired screening-overlap summaries are reported in S1 Appendix Table S11.

The separate screening-only high-missingness stress test removed the highest observed-missingness quartile from each outer-training partition before fresh median/mode screening. Relative to the paired full median/mode screen, the mean top-35 Jaccard overlap was 0.689 (range 0.522–0.892) and the mean full-ranking Spearman correlation was 0.873; common-variable directions again agreed in every comparison. Compared with retained students, the excluded quartile had a mean weighted science score 55.33 points lower and a mean ESCS value about 0.247 points lower. Because model fitting and SHAP were not repeated after quartile exclusion, these results concern screening stability only; they do not establish that the Table 2 performance estimates or SHAP magnitudes and ranks are robust to this sample restriction, identify an MNAR mechanism, or remove residual selection bias.

The subsequent full alternative-imputation analysis re-estimated focal GA-BPNN held-out performance and SHAP using training-derived median/mode imputation in the complete model-development samples. Held-out performance changed in a system- and metric-specific manner: R² was lower than under KNN in CZE, FIN, GBR, and POL and higher in AUS, BEL, IRL, KOR, and LTU; MAE was higher in five systems, while MSE and RMSE were higher in six systems (S1 Appendix Table S13). Under this same alternative-imputation workflow, SHAP magnitudes and ranks varied across systems (S1 Appendix Table S14). Table S14 reports all eight primary recurrent variables—ESCS, HOMEPOS, MATHEFF, ST255Q01JA, ST290Q03WA, ST290Q05WA, ST290Q07WA, and ST290Q09WA. The four sensitivity-supported variables—ESCS, HOMEPOS, MATHEFF, and ST255Q01JA—are the conservative subset that also recurred across the alternative-imputation and high-missingness screening analyses. They do not replace the eight primary variables and are not defined as the four highest-ranked SHAP variables. Together, these results support construct-level recurrence for the sensitivity-supported subset but not exact variable-level membership, attribution-rank, or attribution-magnitude invariance.

### 3.5. SHAP-based attribution heterogeneity

SHAP was used to describe how variables contributed to the output of the focal GA-BPNN model. The results report an equal-system global ranking and a within-system-normalized recurrent-variable heat map. They are conditional model summaries, not causal effects, policy-impact estimates, or independent individual-level predictions.

Fig 3 presents the equal-system global GA-BPNN ranking across 9 × 20 outer holdouts. Each split used DeepExplainer with 256 outer-training background observations and all outer-test students. W_FSTUWT-weighted mean absolute SHAP values were aggregated across plausible values and splits after signed one-hot contributions were recombined at the original-variable level. Table 3 provides plain-language definitions. The corresponding numerical ranking for the 15 highest-ranked variables is reported in S1 Appendix Table S5.

Fig 4 displays within-system-normalized SHAP importance for the eight variables recurrent under the primary KNN top-35 procedure in every outer split of all nine education systems: ESCS, HOMEPOS, MATHEFF, ST255Q01JA, ST290Q03WA, ST290Q05WA, ST290Q07WA, and ST290Q09WA. Differences show model-attribution heterogeneity; recurrence does not imply identical roles or causal effects, and the ST290 item-level variables were sensitive to the missing-data screening procedures. Within-system-normalized mean absolute SHAP values and selection frequencies for these eight variables are reported in S1 Appendix Table S4.

The two displays summarize the behavior of the focal GA-BPNN model across the fixed cohort. They do not establish that the model is systematically superior, that the variables predict an independent outcome, or that the attributed variables are intervention targets.

The architecture-dependence check showed substantial but incomplete agreement between GA-BPNN SHAP and XGBoost TreeSHAP across the 180 paired splits. The fixed-cohort mean top-10 Jaccard overlap was 0.716 (SD 0.123), with system means ranging from 0.609 to 0.841; the mean feature-rank Spearman correlation was 0.824 (SD 0.061), with system means ranging from 0.757 to 0.885 (S1 Appendix Table S15). Repeated patterns across the two architectures provide descriptive cross-architecture context, while exact SHAP magnitudes and ranks remain model-conditional.

## 4. Conclusion and discussion

This study maps recurrent and system-specific survey-weighted descriptive association patterns in PISA 2022 science performance across a fixed, non-probability cohort of nine education systems. The repeated model fits and focal GA-BPNN SHAP summaries show that background, home-resource, and mathematics-learning variables recur across these cases while their model-attribution magnitudes vary. The alternative-imputation analysis showed system- and metric-specific performance changes, and the cross-architecture check showed substantial but incomplete agreement between GA-BPNN SHAP and XGBoost TreeSHAP. The eight primary recurrent variables remain the set displayed in Fig 4, whereas the four sensitivity-supported variables—ESCS, HOMEPOS, MATHEFF, and ST255Q01JA—form a conservative subset supported across the missing-data screening conditions. The contribution is therefore a comparative description of model-dependent association structure, not an individual diagnostic prediction system.

Several constructs recurred across the nine system-level models, including socioeconomic resources, home resources, and mathematics self-beliefs. These are overlapping indicators rather than isolated mechanisms. ESCS represents a broad socioeconomic and cultural-status construct [22,23]. It recurred under marginal Spearman screening but was retained by both regularized models in only five systems, which is compatible with shared information among ESCS, home-resource, parental-background, and learning variables. This pattern does not establish causal effects or the absence of socioeconomic relevance in the other systems.

Recurrent variables also had heterogeneous attribution patterns across education systems [5]. MATHEFF and home-resource variables, for example, differed in their relative contributions across fitted models [3,5,22–28]. Such differences may reflect contextual variation, correlated predictors, questionnaire response processes, and information represented in the PISA conditioning model. They should therefore be interpreted locally and descriptively rather than translated directly into policy interventions.

The framework is useful for identifying where a fitted model shows common versus system-specific association patterns. It does not isolate mechanisms, estimate intervention effects, or validate individual student scores. Any substantive interpretation should be triangulated with theory, external outcomes, and designs suited to causal or individual-level inference.

This study has several limitations. First, plausible values are multiple imputations for population inference, not observed individual outcomes; moreover, the PISA conditioning model includes background information related to candidate predictors. The fitted models may therefore reconstruct information represented in the latent-outcome model and cannot be interpreted as independent predictions of individual achievement. Second, the nine systems are a fixed, non-probability analytical case cohort without a reconstructible system-level inclusion rule; transparent membership reporting does not remove the resulting limit on generalization. Third, missingness was associated with science performance and ESCS. The full alternative-imputation analysis showed system- and metric-specific changes in held-out performance and variation in SHAP magnitude and rank, whereas the high-missingness quartile analysis was screening-only. The eight primary recurrent variables remain the set displayed in Fig 4; the four sensitivity-supported variables—ESCS, HOMEPOS, MATHEFF, and ST255Q01JA—are a conservative subset. Together these analyses do not establish MAR, identify MNAR, eliminate residual selection bias, or establish exact variable-level attribution invariance. Fourth, Table 2 is descriptive and does not establish systematic model superiority. Fifth, SHAP values are conditional on the focal GA-BPNN model, correlated inputs, and the aggregation procedure; cross-architecture agreement with XGBoost was substantial but incomplete. External outcomes and designs suited to causal or individual-level inference are needed before stronger conclusions.

## Supporting information

S1 Appendix Supplementary methods, results, and tables. This appendix includes Supplementary Methods S1-S10, Appendices A-E, and Tables S1-S15.
(DOCX)
