# Supplementary material for: Recurrent and system-specific model-attribution patterns in PISA 2022 science performance across nine education systems
Source: PLoS One. 2026 Sep 18;21(9):e0351534. doi: 10.1371/journal.pone.0351534 (PMC13588497; doi:10.1371/journal.pone.0351534)
Supplement: Supplementary file 1 [file pone.0351534.s001.docx]

**S1 Appendix**

**Recurrent and system-specific model-attribution patterns in PISA 2022 science performance across nine education systems**

Meng Liu^1^ Xin Bai^2^ Xiaohong Li ^3^ Wei Jin^1,*^ Weifeng Jin^3,*^

^1^ School of Artificial Intelligence in Traditional Chinese Medicine, Zhejiang Chinese Medical University, Hangzhou, China

^2^ School of Medical Technology, Zhejiang Chinese Medical University, Hangzhou, China

^3^ School of Pharmaceutical Sciences, Zhejiang Chinese Medical University, Hangzhou, China

Meng Liu: [liumeng@zcmu.edu.cn](mailto:liumeng@zcmu.edu.cn)

Xin Bai: [bai_xinX2011@163.com](mailto:bai_xinX2011@163.com)

Xiaohong Li: [li_xiaoh2005@163.com](mailto:li_xiaoh2005@163.com)

Wei Jin: [jinwei@zcmu.edu.cn](mailto:jinwei@zcmu.edu.cn)

Weifeng Jin: [kim_edu@126.com](mailto:kim_edu@126.com)

*Correspondence E-mail address: jinwei@zcmu.edu.cn (WJ); kim_edu@126.com (WFJ)

# **Supplementary Methods**

## **S1. Survey-weighted summaries and partition-based model evaluation**

Full-sample science means and standard errors combine W_FSTUWT, the ten plausible values, and all 80 Fay-adjusted BRR replicate weights. Repeated train/test analyses instead use W_FSTUWT-weighted metrics for each plausible value and report the mean and split-to-split SD across 20 school-grouped holdouts. The BRR weights are not used to claim design-consistent uncertainty within partial holdout samples.

$${R^{2}}_{w}=1-\frac{{\sum_{i=1}^{N} w_{i}\left( y_{i}-\hat{y}_{i} \right)}^{2}}{{\sum_{i=1}^{N} w_{i}\left( y_{i}-\bar{y}_{w} \right)}^{2}}$$

(1)

$$\mathrm{MAE}_{w}=\frac{\sum_{i=1}^{N} w_{i}\left| y_{i}-\hat{y}_{i} \right|}{\sum_{i=1}^{N} w_{i}}$$

(2)

$$\mathrm{MSE}_{w}=\frac{\sum_{i=1}^{N} w_{i}{(y_{i}-\hat{y}_{i})}^{2}}{\sum_{i=1}^{N} w_{i}}$$

(3)

$$V_{W}=\frac{1}{M}\sum_{m=1}^{M} V_{BRR}^{(m)}$$

(4)

$$V_{BRR}^{(m)}=\frac{1}{G{(1-k)}^{2}}\sum_{g=1}^{G} {(\theta_{mg}-\theta_{m})}^{2}$$

(5)

$$V_{B}=\frac{1}{M-1}\sum_{m=1}^{M} {(\theta_{m}-\bar{\theta})}^{2}$$

(6)

$$\mathrm{SE}=\sqrt{V_{W}+(1+\frac{1}{M})V_{B}}$$

(7)

## **S2. Leakage control and model-comparison protocol**

All outer and inner partitions used CNTSCHID as the grouping variable so that students from one school remained in one partition. Training-only operations comprised system-specific missingness filtering, KNN imputation, weighted Spearman screening, categorical encoding, standardization, model selection, and optimization-based initialization. The held-out partition was not used for any training decision. These repeated partitions summarize stability across sampled schools within an education system; they do not validate an independent individual outcome.

## **S3. LASSO and Elastic Net sensitivity analysis**

The LASSO and Elastic Net analysis was an exploratory sensitivity check on feature retention within the model-ready matrix. Models were fitted on training data only with W_FSTUWT-weighted loss. The mean of the ten plausible values was used solely as a computational target for this sensitivity check and is not interpreted as an observed individual score. Regularization strength was selected within the training data.

The sensitivity analysis retained MATHEFF, ST256Q03JA, ST256Q06JA, ST290Q05WA, and ST290Q09WA in all nine education systems under both LASSO and Elastic Net. ST290Q01WA was retained in eight of nine systems under both methods. ESCS was retained by both regularized models in Australia, Finland, Ireland, Korea, and Lithuania, but not in Belgium, the Czech Republic, the United Kingdom, or Poland. These results indicate sensitivity to embedded regularization within the model-ready feature matrix, not as a replacement for the primary weighted Spearman screening workflow or as evidence that ESCS is irrelevant in the four systems.

## **S4. Shared BPNN architecture and training procedure**

All neural-network pipelines used a feed-forward BPNN with one hidden layer, sigmoid activation, 40% dropout, and one continuous output. System-specific hidden widths were AUS=14, BEL=12, CZE=12, FIN=14, GBR=15, IRL=10, KOR=13, LTU=12, and POL=10. Final fitting used W_FSTUWT-weighted mean-squared-error loss, Adam, learning rate 0.005, weight decay 0.001, batch size 1,024, at most 400 epochs, and early-stopping patience 35. GA, PSO, and SA were initialization stages before the same backpropagation procedure.

## **S5. GA-BPNN optimization details**

In the GA-BPNN pipeline, a chromosome represented the initial weights and biases of the BPNN. The genetic algorithm searched for initial network parameters before final backpropagation training. The current implementation used a population size of 40 and 20 generations. During fitness evaluation, each candidate network was briefly trained for 20 epochs on the training subset using a batch size of 2048, learning rate of 0.005, weight decay of 0.001, and dropout of 0.4, and was then evaluated on the internal-validation subset. Parent selection used tournament selection with k = 3. Offspring were generated using simulated binary crossover (eta = 15) and Gaussian mutation (mutation rate = 0.15; mutation scale = 0.1). The parameter bound was [-2.0, 2.0]. The best initial-weight configuration was then used to initialize the final BPNN training procedure. Candidate chromosomes were ranked using the following composite validation fitness:

$$F = 0.40R_{val}^{2} - 0.30{MAE}_{val} - 0.30{RMSE}_{val} - 0.15|{MSE}_{val} - {MSE}_{train}|$$

(8)

Here, all metrics were calculated with sampling weights on training-only standardized plausible-value targets and averaged across the ten plausible values. The fitness function rewards higher validation accuracy while penalizing a large absolute gap between training and validation MSE.

## **S6. PSO-BPNN optimization details**

In the PSO-BPNN pipeline, each particle represented a candidate set of initial BPNN weights and biases. The PSO implementation used a swarm size of 40 and 20 iterations. The inertia weight was 0.7, and the cognitive and social coefficients were both 1.5. Initial velocities were set to zero, and the parameter bound was [-2.0, 2.0]. Particle velocities and positions were updated as follows:

$$\left\{ \begin{aligned} v_{i,j}\left( t+1 \right)=\omega\cdot v_{i,j}\left( t \right)+c_{1}\cdot r_{1}\cdot\left( p_{i,j}\left( t \right)-x_{i,j}\left( t \right) \right)+c_{2}\cdot r_{2}\cdot\left( g_{j}\left( t \right)-x_{i,j}\left( t \right) \right) \\ x_{i,j}\left( t+1 \right)=x_{i,j}\left( t \right)+v_{i,j}\left( t+1 \right) \end{aligned} \right.$$

(9)

Each candidate was briefly trained using the same 20-epoch candidate-training settings specified in S5. The composite validation fitness defined in S5 was calculated on the internal-validation subset and used to update the personal-best and global-best positions. The best particle was then used as the initial configuration for final BPNN training.

## **S7. SA-BPNN optimization details**

In the SA-BPNN pipeline, a state represented a candidate set of BPNN weights and biases. The simulated-annealing procedure perturbed the current state and accepted or rejected candidate states according to the Metropolis criterion. The implementation used an initial temperature of 1.0, a final temperature of 0.1, a cooling coefficient of 0.9, a Markov-chain length of 10 at each temperature, and a perturbation step size of 0.1. Candidate states were bounded to [-2.0, 2.0]. As with GA and PSO, each candidate was briefly trained using the settings specified in S5 and assessed using the composite validation fitness before the selected initialization was passed to final BPNN training.

## **S8. Hyperparameter budgets and search spaces**

The seven pipelines used shared partitions and documented settings. The BPNN variants shared the architecture and final fitting configuration; GA, PSO, and SA differed only in initialization. RF, XGBoost, and SVR used the settings in Table S2. Comparisons are descriptive for the fixed cohort and do not constitute a formal test of systematic superiority.

## **S9. Cross-system global SHAP attribution analysis**

The global SHAP analysis used DeepExplainer for the focal GA-BPNN in 20 school-grouped outer splits per education system. For every outer split and plausible value, 256 observations were sampled without replacement from the corresponding outer-training partition with probabilities proportional to W_FSTUWT; the sampling seed was the outer-split seed plus 314159. SHAP values were computed for all students in each outer-test set. Signed one-hot SHAP values were summed to the original-variable level before absolute values were taken. Split-level global importance was the W_FSTUWT-weighted mean absolute SHAP value across held-out students. Values were averaged across plausible values and splits, with zero assigned when a variable was not selected; education systems then received equal weight.

The completed analysis covered 180 system-split SHAP outputs. Supplementary Table S4 reports within-system-normalized importance and selection frequency for the eight variables recurrent under the primary KNN top-35 procedure. Supplementary Table S5 reports the global ranking and selection frequencies for the 15 highest-ranked variables. These results are focal GA-BPNN attribution summaries and are not claimed to be invariant across model classes.

## **S10. Residual diagnostics as descriptive checks**

Residual diagnostics are descriptive checks of fitted-model error structure. They were not used for pipeline selection, optimization, or ranking. Held-out model fit is reported with W_FSTUWT-weighted R², MAE, MSE, and RMSE and split-to-split SD; BRR-based design-consistent inference is not claimed for partial holdouts.

# **Supplementary Results**

## **Appendix A. Missingness diagnostics and complete-case feasibility**

Missingness was calculated from the raw PISA 2022 student file using the frozen 114-variable union of the nine 35-variable model-ready feature sets. The public repository separately provides the 488-variable candidate eligibility registry used for screening. All 29 Level 3 systems and their inclusion indicators are reported in Table S10. The nine-system cohort had lower mean missingness than the other Level 3 systems on average, but several excluded systems had lower missingness than included systems; therefore, missingness is a cohort descriptor, not a selection cutoff. Complete-case deletion across the 35 screened variables retained almost no students in several systems and was not viable as a primary analysis.

## **Appendix B. Missingness-burden diagnostic**

Students were grouped by quartiles of missingness burden across screened variables. The highest-missingness group often had lower science plausible values and lower ESCS than the lowest-missingness group. This diagnostic demonstrates sensitivity of the analytical sample to missingness burden but does not establish a missing-at-random mechanism or remove selection bias.

## **Appendix C. Feature-threshold sensitivity**

The recurrent-variable screening audit was repeated under top-25, top-35, and top-45 retention thresholds. A variable was counted as recurrent in an education system only when it was selected in all 20 school-grouped outer splits for that system. Four variables were recurrent in all nine systems at top-25: ESCS, MATHEFF, ST290Q05WA, and ST290Q09WA. At top-35, HOMEPOS, ST255Q01JA, ST290Q03WA, and ST290Q07WA also became recurrent in all nine systems. At the top-45 threshold, IC173Q04JA and ST290Q01WA became recurrent in all nine systems. The full audit is reported in Supplementary Table S9.

## **Appendix D. Missing-data sensitivity analysis**

The screening-only component compared primary KNN screening with a fresh training-derived median/mode implementation across the nine education systems and 20 school-grouped outer-training partitions per system. The mean top-35 Jaccard overlap was 0.504 (range 0.346-0.750), the mean full-ranking Spearman correlation was 0.783, and the mean sign agreement among common top-35 variables was 1.000. ESCS, HOMEPOS, MATHEFF, and ST255Q01JA remained selected in every split of all nine systems under both procedures. No focal-model performance or SHAP analysis was re-estimated for this screening-only comparison.

The separate high-missingness stress test was also restricted to feature screening. The highest observed-missingness quartile was defined within each outer-training partition, removed before fresh median/mode screening, and compared with the paired full median/mode screen; outer-test data remained unchanged. The mean top-35 Jaccard overlap was 0.689 (range 0.522-0.892), the mean full-ranking Spearman correlation was 0.873, and the common-variable sign agreement was 1.000. Compared with retained students, the excluded quartile had a mean weighted science score 55.33 points lower and a mean ESCS value about 0.247 points lower. This stress test does not identify an MNAR mechanism, eliminate residual selection bias, or establish the robustness of model performance or SHAP results to the quartile exclusion.

## **Appendix E. Cohort definition across all Level 3 education systems**

Table S10 reports all 29 Level 3 systems using the frozen 114-variable registry. The analyzed nine-system cohort is a fixed, non-probability analytical case cohort with no reconstructible system-level missingness cutoff. The table makes membership transparent and auditable without converting the historical choice into a reproducible sampling rule.

# **Supplementary Tables**

**Supplementary Table S1. Hyperparameter specifications and architectural details for the BPNN baseline and hybrid optimization models.**

| **Model** | **Component / Category** | **Parameter (Variable)** | **Value / Setting** |
| --- | --- | --- | --- |
| Hybrid Models (Common Settings) | Training Strategy | Base Learning Rate | 0.005 |
|  |  | Batch Size | 1024 |
|  |  | Dropout Rate | 0.4 |
|  |  | L2 Regularization | 0.001 |
|  | Final BPNN Training | Maximum Epochs | 400 |
|  |  | Early-stopping Patience | 35 |
|  | Objective Function | Composite Validation Fitness | $F = 0.40 R_{val}^{2} - 0.30 {MAE}_{val} - 0.30 {RMSE}_{val} - 0.15 \vert{MSE}_{val} - {MSE}_{train}\vert$ |
|  | Model Configuration | Hidden Neurons | See Supplementary Table S3 |
| GA-BPNN | Optimization Algorithm (GA) | Population Size | 40 |
|  |  | Generations | 20 |
|  |  | Mutation Rate | 0.15 |
| PSO-BPNN | Optimization Algorithm (PSO) | Particle Number | 40 |
|  |  | Iterations | 20 |
|  |  | Acceleration Coefficients | c1 = 1.5; c2 = 1.5 |
|  |  | Initial Velocity | 0.0 |
| SA-BPNN | Optimization Algorithm (SA) | Initial Temperature | 1.0 |
|  |  | Final Temperature | 0.1 |
|  |  | Cooling Rate | 0.9 |
|  |  | Markov-chain Length | 10 |
|  |  | Perturbation Step Size | 0.1 |

**Supplementary Table S2. Hyperparameter settings and search spaces for tree-based (XGBoost, Random Forest) and regression (SVR) models.**

| **Model** | **Component / Category** | **Parameter (Variable)** | **Optimal Value** | **Description / Search Space** |
| --- | --- | --- | --- | --- |
| XGBoost | Boosting Strategy | Number of Trees (n_estimators) | 1000 | Total number of gradient boosted trees |
|  |  | Learning Rate (learning_rate) | 0.02 | Step size shrinkage to prevent overfitting |
|  | Tree Structure | Max Tree Depth (max_depth) | 4 | Maximum depth of a tree |
|  |  | Subsample Ratio (subsample) | 0.7 | Fraction of observations to be randomly sampled |
|  |  | Column Subsample (colsample_bytree) | 0.7 | Fraction of columns to be randomly sampled |
|  | Regularization | Min Split Loss (gamma) | 1.0 | Minimum loss reduction required for a split |
|  |  | L1 Regularization (reg_alpha) | 0.5 | L1 regularization term on weights |
|  |  | L2 Regularization (reg_lambda) | 0.5 | L2 regularization term on weights |
| SVR | Kernel Specification | Kernel Function | RBF | Radial Basis Function |
|  | Hyperparameters | Regularization (C) | 0.1 | Grid Search: [0.1, 1, 10, 50, 100] |
|  |  | Kernel Coefficient (Gamma) | 0.01 | Grid Search: [0.01, 0.1, 1, 'scale', 'auto'] |
|  | Validation Strategy | Metric / Method | MSE | 5-fold Cross Validation |
| Random Forest | Ensemble Strategy | Number of Trees (n_estimators) | 500 | Total number of trees in the forest |
|  | Tree Structure | Max Tree Depth (max_depth) | 7 | Maximum depth of the tree |
|  |  | Min Samples Leaf (min_samples_leaf) | 20 | Minimum samples required at a leaf node |
|  |  | Max Features (max_features) | 'sqrt' | Number of features to consider per split |

**Supplementary Table S3. Hidden-layer widths used by the BPNN pipelines across education systems.**

| **Education system** | **Hidden Neurons** |
| --- | --- |
| Australia | 14 |
| Belgium | 12 |
| Czech Republic | 12 |
| Finland | 14 |
| Ireland | 10 |
| Korea | 13 |
| Lithuania | 12 |
| Poland | 10 |
| United Kingdom | 15 |

**Supplementary Table S4. Within-system-normalized mean absolute SHAP importance and selection frequency for the eight variables recurrent under the top-35 procedure.**

| **Education system** | **Variable** | **Normalized mean absolute SHAP** | **Selection frequency** |
| --- | --- | --- | --- |
| AUS | ESCS | 0.0077 | 1.00 |
| AUS | HOMEPOS | 0.0260 | 1.00 |
| AUS | MATHEFF | 0.0545 | 1.00 |
| AUS | ST255Q01JA | 0.0393 | 1.00 |
| AUS | ST290Q03WA | 0.0228 | 1.00 |
| AUS | ST290Q05WA | 0.0293 | 1.00 |
| AUS | ST290Q07WA | 0.0082 | 1.00 |
| AUS | ST290Q09WA | 0.0298 | 1.00 |
| BEL | ESCS | 0.0135 | 1.00 |
| BEL | HOMEPOS | 0.0288 | 1.00 |
| BEL | MATHEFF | 0.0513 | 1.00 |
| BEL | ST255Q01JA | 0.0342 | 1.00 |
| BEL | ST290Q03WA | 0.0268 | 1.00 |
| BEL | ST290Q05WA | 0.0122 | 1.00 |
| BEL | ST290Q07WA | 0.0072 | 1.00 |
| BEL | ST290Q09WA | 0.0138 | 1.00 |
| CZE | ESCS | 0.0169 | 1.00 |
| CZE | HOMEPOS | 0.0380 | 1.00 |
| CZE | MATHEFF | 0.0400 | 1.00 |
| CZE | ST255Q01JA | 0.0383 | 1.00 |
| CZE | ST290Q03WA | 0.0286 | 1.00 |
| CZE | ST290Q05WA | 0.0118 | 1.00 |
| CZE | ST290Q07WA | 0.0085 | 1.00 |
| CZE | ST290Q09WA | 0.0195 | 1.00 |
| FIN | ESCS | 0.0123 | 1.00 |
| FIN | HOMEPOS | 0.0367 | 1.00 |
| FIN | MATHEFF | 0.0254 | 1.00 |
| FIN | ST255Q01JA | 0.0416 | 1.00 |
| FIN | ST290Q03WA | 0.0130 | 1.00 |
| FIN | ST290Q05WA | 0.0099 | 1.00 |
| FIN | ST290Q07WA | 0.0060 | 1.00 |
| FIN | ST290Q09WA | 0.0225 | 1.00 |
| GBR | ESCS | 0.0189 | 1.00 |
| GBR | HOMEPOS | 0.0216 | 1.00 |
| GBR | MATHEFF | 0.0491 | 1.00 |
| GBR | ST255Q01JA | 0.0362 | 1.00 |
| GBR | ST290Q03WA | 0.0226 | 1.00 |
| GBR | ST290Q05WA | 0.0124 | 1.00 |
| GBR | ST290Q07WA | 0.0105 | 1.00 |
| GBR | ST290Q09WA | 0.0262 | 1.00 |
| IRL | ESCS | 0.0134 | 1.00 |
| IRL | HOMEPOS | 0.0167 | 1.00 |
| IRL | MATHEFF | 0.0636 | 1.00 |
| IRL | ST255Q01JA | 0.0462 | 1.00 |
| IRL | ST290Q03WA | 0.0226 | 1.00 |
| IRL | ST290Q05WA | 0.0176 | 1.00 |
| IRL | ST290Q07WA | 0.0093 | 1.00 |
| IRL | ST290Q09WA | 0.0243 | 1.00 |
| KOR | ESCS | 0.0108 | 1.00 |
| KOR | HOMEPOS | 0.0393 | 1.00 |
| KOR | MATHEFF | 0.0777 | 1.00 |
| KOR | ST255Q01JA | 0.0263 | 1.00 |
| KOR | ST290Q03WA | 0.0211 | 1.00 |
| KOR | ST290Q05WA | 0.0117 | 1.00 |
| KOR | ST290Q07WA | 0.0108 | 1.00 |
| KOR | ST290Q09WA | 0.0212 | 1.00 |
| LTU | ESCS | 0.0237 | 1.00 |
| LTU | HOMEPOS | 0.0099 | 1.00 |
| LTU | MATHEFF | 0.0495 | 1.00 |
| LTU | ST255Q01JA | 0.0415 | 1.00 |
| LTU | ST290Q03WA | 0.0249 | 1.00 |
| LTU | ST290Q05WA | 0.0150 | 1.00 |
| LTU | ST290Q07WA | 0.0123 | 1.00 |
| LTU | ST290Q09WA | 0.0323 | 1.00 |
| POL | ESCS | 0.0227 | 1.00 |
| POL | HOMEPOS | 0.0169 | 1.00 |
| POL | MATHEFF | 0.0545 | 1.00 |
| POL | ST255Q01JA | 0.0479 | 1.00 |
| POL | ST290Q03WA | 0.0424 | 1.00 |
| POL | ST290Q05WA | 0.0123 | 1.00 |
| POL | ST290Q07WA | 0.0139 | 1.00 |
| POL | ST290Q09WA | 0.0189 | 1.00 |

**Supplementary Table S5. Global GA-BPNN SHAP ranking for the 15 highest-ranked PISA variables across the nine education systems. Global importance is the equal-system mean of system-level unconditional weighted mean absolute SHAP values.**

| **Variable** | **Equal-system mean absolute SHAP** | **Mean split selection frequency** | **Systems selected** |
| --- | --- | --- | --- |
| IC173Q04JA | 7.929 | 0.983 | 9/9 |
| MATHEFF | 6.792 | 1.000 | 9/9 |
| ST255Q01JA | 5.076 | 1.000 | 9/9 |
| ST294Q05JA | 5.014 | 0.611 | 6/9 |
| IC180Q08JA | 4.609 | 0.839 | 8/9 |
| ST256Q03JA | 3.766 | 0.600 | 6/9 |
| IC183Q01JA | 3.637 | 0.783 | 9/9 |
| HOMEPOS | 3.453 | 1.000 | 9/9 |
| ST290Q03WA | 3.242 | 1.000 | 9/9 |
| ST294Q04JA | 3.220 | 0.650 | 6/9 |
| ST290Q09WA | 3.035 | 1.000 | 9/9 |
| ST275Q05WA | 2.963 | 0.706 | 7/9 |
| HISEI | 2.918 | 0.861 | 8/9 |
| ST303Q05JA | 2.815 | 0.411 | 6/9 |
| ST275Q07WA | 2.737 | 0.772 | 8/9 |

**Supplementary Table S6. Missingness diagnostics by performance level and inclusion status.**

| **Education system** | **IncludedInStudy** | **Systems** | **TotalN** | **MeanScience** | **MeanVarMissing** | **MedianVarMissing** |
| --- | --- | --- | --- | --- | --- | --- |
| Level 1 (<410) | False | 25 | 178489 | 380.9 | 0.448 | 0.437 |
| Level 2 (410-484) | False | 25 | 190288 | 443.7 | 0.354 | 0.250 |
| Level 3 (484-559) | False | 20 | 159676 | 504.5 | 0.319 | 0.173 |
| Level 3 (484-559) | True | 9 | 78685 | 502.4 | 0.274 | 0.153 |
| Level 4 (>=559) | False | 1 | 6606 | 561.4 | 0.249 | 0.034 |

**Supplementary Table S7. Missingness-burden diagnostic by retained education system.**

| **Education system** | **N** | **MeanMissingFraction** | **ScienceQ4minusQ1** | **ESCSQ4minusQ1** | **CorrMissingScience** | **CorrMissingESCS** |
| --- | --- | --- | --- | --- | --- | --- |
| AUS | 13437 | 0.202 | -64.0 | -0.277 | -0.242 | -0.085 |
| BEL | 8286 | 0.193 | -83.5 | -0.539 | -0.339 | -0.192 |
| CZE | 8460 | 0.206 | -65.8 | -0.401 | -0.275 | -0.176 |
| FIN | 10239 | 0.280 | -85.9 | -0.188 | -0.352 | -0.119 |
| GBR | 12972 | 0.313 | -67.3 | -0.286 | -0.207 | -0.069 |
| IRL | 5569 | 0.159 | -64.0 | -0.286 | -0.324 | -0.139 |
| KOR | 6454 | 0.341 | -23.5 | -0.123 | -0.147 | -0.045 |
| LTU | 7257 | 0.183 | -65.2 | -0.286 | -0.300 | -0.076 |
| POL | 6011 | 0.197 | -72.0 | -0.232 | -0.331 | -0.084 |

**Supplementary Table S8. Complete-case feasibility diagnostic across the system-specific top-35 screened variables.**

| **Education system** | **N** | **System top-35 features** | **CompleteCaseN** | **CompleteCasePct** | **MeanFeatureMissingRateTop35** | **MedianFeatureMissingRateTop35** |
| --- | --- | --- | --- | --- | --- | --- |
| AUS | 13437 | 35 | 2 | 0.0001 | 0.202 | 0.124 |
| BEL | 8286 | 35 | 30 | 0.0036 | 0.193 | 0.123 |
| CZE | 8460 | 35 | 3 | 0.0004 | 0.206 | 0.129 |
| FIN | 10239 | 35 | 0 | 0.0000 | 0.280 | 0.252 |
| GBR | 12972 | 35 | 0 | 0.0000 | 0.313 | 0.288 |
| IRL | 5569 | 35 | 0 | 0.0000 | 0.159 | 0.100 |
| KOR | 6454 | 35 | 0 | 0.0000 | 0.341 | 0.455 |
| LTU | 7257 | 35 | 3 | 0.0004 | 0.183 | 0.101 |
| POL | 6011 | 35 | 0 | 0.0000 | 0.197 | 0.115 |

**Supplementary Table S9. Threshold-sensitivity audit for variables selected in all 20 outer splits within an education system under top-25, top-35, and top-45 weighted Spearman screening. Values are the number of education systems out of nine.**

| **Variable** | **Top-25 systems** | **Top-35 systems** | **Top-45 systems** | **Classification** |
| --- | --- | --- | --- | --- |
| ESCS | 9/9 | 9/9 | 9/9 | Threshold-stable core variable |
| MATHEFF | 9/9 | 9/9 | 9/9 | Threshold-stable core variable |
| ST290Q05WA | 9/9 | 9/9 | 9/9 | Threshold-stable core variable |
| ST290Q09WA | 9/9 | 9/9 | 9/9 | Threshold-stable core variable |
| HOMEPOS | 7/9 | 9/9 | 9/9 | Top-35 threshold-sensitive extension |
| ST255Q01JA | 8/9 | 9/9 | 9/9 | Top-35 threshold-sensitive extension |
| ST290Q03WA | 8/9 | 9/9 | 9/9 | Top-35 threshold-sensitive extension |
| ST290Q07WA | 8/9 | 9/9 | 9/9 | Top-35 threshold-sensitive extension |
| IC173Q04JA | 5/9 | 8/9 | 9/9 | Top-45 threshold-sensitive extension |
| ST290Q01WA | 7/9 | 8/9 | 9/9 | Top-45 threshold-sensitive extension |

**Supplementary Table S10. Weighted mean science performance, mean variable missingness, and cohort inclusion for all Level 3 PISA 2022 education systems.**

| **System code** | **Student N** | **Weighted mean science** | **Mean variable missingness** | **Included** |
| --- | --- | --- | --- | --- |
| MAC | 4384 | 543.10 | 0.158 | No |
| KOR | 6454 | 527.82 | 0.182 | Yes |
| IRL | 5569 | 503.85 | 0.187 | Yes |
| HKG | 5907 | 520.42 | 0.212 | No |
| TAP | 5857 | 537.38 | 0.246 | No |
| EST | 6392 | 525.81 | 0.249 | No |
| LVA | 5373 | 493.84 | 0.256 | No |
| JPN | 5760 | 546.63 | 0.259 | No |
| AUS | 13437 | 507.00 | 0.273 | Yes |
| LTU | 7257 | 484.46 | 0.274 | Yes |
| POL | 6011 | 499.16 | 0.274 | Yes |
| HUN | 6198 | 485.89 | 0.278 | No |
| SVN | 6721 | 499.96 | 0.279 | No |
| ESP | 30800 | 484.53 | 0.281 | No |
| CZE | 8460 | 497.74 | 0.286 | Yes |
| BEL | 8286 | 490.58 | 0.304 | Yes |
| AUT | 6151 | 491.27 | 0.306 | No |
| FIN | 10239 | 510.96 | 0.320 | Yes |
| CHE | 6829 | 502.52 | 0.325 | No |
| USA | 4552 | 499.41 | 0.333 | No |
| SWE | 6072 | 493.55 | 0.336 | No |
| DEU | 6116 | 492.43 | 0.349 | No |
| PRT | 6793 | 484.37 | 0.359 | No |
| DNK | 6200 | 493.82 | 0.361 | No |
| GBR | 12972 | 499.67 | 0.365 | Yes |
| NLD | 5046 | 488.32 | 0.439 | No |
| NZL | 4682 | 504.13 | 0.439 | No |
| FRA | 6770 | 487.23 | 0.447 | No |
| CAN | 23073 | 515.02 | 0.463 | No |

**Supplementary Table S11. Missing-data feature-screening sensitivity summary.**

| **Comparison** | **Mean top-35 Jaccard** | **Range** | **Mean ranking Spearman** | **Common top-35 sign agreement** |
| --- | --- | --- | --- | --- |
| KNN vs fresh median/mode | 0.504 | 0.346-0.750 | 0.783 | 1.000 |
| Full vs Q4-excluded median/mode | 0.689 | 0.522-0.892 | 0.873 | 1.000 |

**Supplementary Table S12. Complete descriptive held-out MAE, MSE, and RMSE summary for seven pipelines across nine education systems.**

| **Model** | **AUS** | **BEL** | **CZE** | **FIN** | **IRL** | **KOR** | **LTU** | **POL** | **GBR** |
| --- | --- | --- | --- | --- | --- | --- | --- | --- | --- |
| **Panel A. Held-out MAE, mean (SD)** |  |  |  |  |  |  |  |  |  |
| GA-BPNN | 60.639 (1.027) | 51.531 (1.226) | 55.832 (1.289) | 60.649 (0.869) | 50.210 (1.364) | 65.877 (1.680) | 50.484 (1.418) | 56.139 (1.159) | 63.267 (1.316) |
| PSO-BPNN | 61.239 (1.015) | 52.537 (1.354) | 56.233 (1.332) | 61.346 (0.858) | 50.417 (1.235) | 66.843 (1.712) | 50.472 (1.507) | 56.178 (1.195) | 64.284 (1.232) |
| SA-BPNN | 61.628 (1.025) | 52.537 (1.404) | 56.827 (1.212) | 61.653 (0.850) | 50.331 (1.266) | 68.576 (1.561) | 50.682 (1.474) | 56.390 (1.166) | 64.364 (1.266) |
| BPNN | 62.647 (1.032) | 54.547 (1.348) | 57.457 (1.341) | 63.676 (0.804) | 53.247 (1.367) | 66.901 (1.694) | 50.975 (1.400) | 56.291 (1.158) | 64.268 (1.254) |
| XGBoost | 62.293 (1.002) | 54.941 (1.358) | 57.112 (1.145) | 62.182 (0.760) | 52.871 (1.099) | 66.147 (1.964) | 50.825 (1.547) | 56.432 (1.175) | 64.599 (1.252) |
| Random Forest | 63.703 (1.158) | 54.338 (1.330) | 57.930 (1.582) | 63.216 (0.754) | 53.754 (1.140) | 66.287 (1.545) | 52.683 (1.584) | 57.096 (1.091) | 66.560 (1.388) |
| SVR | 63.901 (1.089) | 55.961 (1.720) | 58.767 (1.231) | 63.358 (0.880) | 51.732 (1.216) | 70.049 (1.994) | 52.198 (1.647) | 58.780 (1.193) | 66.867 (1.381) |
| **Panel B. Held-out MSE, mean (SD)** |  |  |  |  |  |  |  |  |  |
| GA-BPNN | 5,828.056 (225.251) | 4,182.788 (207.951) | 4,793.547 (180.719) | 5,970.440 (144.214) | 4,013.636 (168.427) | 7,114.637 (312.346) | 4,178.627 (217.586) | 4,966.782 (201.297) | 6,317.576 (243.341) |
| PSO-BPNN | 5,984.343 (196.350) | 4,314.022 (205.034) | 4,904.996 (196.624) | 6,116.756 (142.925) | 4,088.828 (172.316) | 7,317.675 (316.553) | 4,071.487 (205.525) | 5,174.369 (202.811) | 6,492.872 (243.091) |
| SA-BPNN | 5,982.381 (209.296) | 4,340.660 (198.821) | 4,904.746 (181.630) | 6,138.467 (149.804) | 4,042.673 (166.330) | 7,842.146 (339.170) | 4,283.965 (218.435) | 5,175.125 (187.585) | 6,487.659 (260.928) |
| BPNN | 6,141.402 (215.446) | 4,584.157 (201.055) | 5,050.694 (190.958) | 6,229.011 (156.557) | 4,138.621 (162.705) | 7,333.358 (327.630) | 4,310.034 (213.021) | 5,160.729 (207.610) | 6,552.046 (244.790) |
| XGBoost | 6,069.195 (163.586) | 4,443.361 (215.758) | 5,093.147 (167.618) | 6,141.545 (141.889) | 4,040.069 (171.305) | 7,218.237 (406.508) | 4,251.572 (229.811) | 4,980.651 (186.492) | 6,476.441 (197.392) |
| Random Forest | 6,419.471 (217.113) | 4,615.935 (243.108) | 5,169.752 (242.406) | 6,541.583 (157.316) | 4,366.354 (155.201) | 7,428.624 (299.855) | 4,541.188 (241.998) | 5,166.941 (176.693) | 6,950.137 (316.176) |
| SVR | 6,517.024 (216.384) | 4,947.266 (254.280) | 5,311.856 (211.706) | 6,288.301 (143.002) | 4,190.840 (181.003) | 7,722.672 (447.574) | 4,332.493 (202.741) | 5,640.465 (202.121) | 7,052.196 (272.372) |
| **Panel C. Held-out RMSE, mean (SD)** |  |  |  |  |  |  |  |  |  |
| GA-BPNN | 76.327 (1.312) | 64.648 (1.486) | 69.213 (1.466) | 77.251 (0.880) | 63.316 (1.558) | 84.322 (1.928) | 64.612 (1.417) | 70.457 (1.415) | 79.461 (1.711) |
| PSO-BPNN | 77.338 (1.208) | 65.660 (1.468) | 70.011 (1.392) | 78.200 (0.895) | 63.919 (1.461) | 85.515 (2.156) | 63.776 (1.469) | 71.907 (1.362) | 80.553 (1.609) |
| SA-BPNN | 77.322 (1.254) | 65.854 (1.433) | 70.006 (1.357) | 78.337 (0.991) | 63.551 (1.429) | 88.523 (1.955) | 65.421 (1.633) | 71.910 (1.365) | 80.526 (1.509) |
| BPNN | 78.347 (1.269) | 67.676 (1.535) | 71.043 (1.452) | 78.912 (0.945) | 64.309 (1.525) | 85.602 (1.909) | 65.630 (1.458) | 71.816 (1.473) | 80.919 (1.616) |
| XGBoost | 77.888 (1.130) | 66.623 (1.645) | 71.346 (1.282) | 78.355 (0.963) | 63.541 (1.396) | 84.925 (1.993) | 65.170 (1.846) | 70.549 (1.325) | 80.460 (1.301) |
| Random Forest | 80.104 (1.328) | 67.911 (1.668) | 71.878 (1.709) | 80.863 (0.935) | 66.052 (1.184) | 86.170 (1.721) | 67.365 (1.691) | 71.867 (1.251) | 83.339 (1.606) |
| SVR | 80.710 (1.195) | 70.304 (1.634) | 72.855 (1.469) | 79.291 (0.892) | 64.718 (1.264) | 87.845 (2.279) | 65.796 (1.621) | 75.077 (1.371) | 83.955 (1.668) |

*Note: Values are mean (SD) across 20 repeated school-grouped outer splits. The ten plausible-value-specific metrics were combined to obtain each split-level summary.*

Abbreviations: AUS, Australia; BEL, Belgium; CZE, Czech Republic; FIN, Finland; IRL, Ireland; KOR, Korea; LTU, Lithuania; POL, Poland; GBR, United Kingdom.

**Supplementary Table S13. GA-BPNN held-out performance under primary KNN and alternative training-only median/mode imputation.**

| **Education system** | **Metric** | **KNN mean (SD)** | **Median/mode mean (SD)** |
| --- | --- | --- | --- |
| AUS | R² | 0.501 (0.018) | 0.509 (0.021) |
| AUS | MAE | 60.639 (1.027) | 60.48 (0.84) |
| AUS | MSE | 5828.056 (225.251) | 5797.87 (166.98) |
| AUS | RMSE | 76.327 (1.312) | 76.13 (1.10) |
| BEL | R² | 0.565 (0.024) | 0.568 (0.023) |
| BEL | MAE | 51.531 (1.226) | 51.39 (0.87) |
| BEL | MSE | 4182.788 (207.951) | 4214.05 (146.09) |
| BEL | RMSE | 64.648 (1.486) | 64.67 (1.32) |
| CZE | R² | 0.507 (0.022) | 0.496 (0.024) |
| CZE | MAE | 55.832 (1.289) | 55.67 (0.90) |
| CZE | MSE | 4793.547 (180.719) | 4851.63 (172.09) |
| CZE | RMSE | 69.213 (1.466) | 70.06 (1.05) |
| FIN | R² | 0.495 (0.012) | 0.486 (0.015) |
| FIN | MAE | 60.649 (0.869) | 61.17 (1.32) |
| FIN | MSE | 5970.440 (144.214) | 5992.40 (247.86) |
| FIN | RMSE | 77.251 (0.880) | 77.74 (1.61) |
| GBR | R² | 0.456 (0.019) | 0.448 (0.027) |
| GBR | MAE | 63.267 (1.316) | 65.02 (1.49) |
| GBR | MSE | 6317.576 (243.341) | 6470.83 (336.87) |
| GBR | RMSE | 79.461 (1.711) | 80.65 (1.84) |
| IRL | R² | 0.516 (0.017) | 0.519 (0.015) |
| IRL | MAE | 50.210 (1.364) | 50.28 (1.09) |
| IRL | MSE | 4013.636 (168.427) | 4017.11 (170.61) |
| IRL | RMSE | 63.316 (1.558) | 63.36 (1.42) |
| KOR | R² | 0.378 (0.029) | 0.383 (0.035) |
| KOR | MAE | 65.877 (1.680) | 64.36 (2.33) |
| KOR | MSE | 7114.637 (312.346) | 7034.30 (298.48) |
| KOR | RMSE | 84.322 (1.928) | 83.97 (3.63) |
| LTU | R² | 0.496 (0.025) | 0.507 (0.022) |
| LTU | MAE | 50.484 (1.418) | 50.73 (1.27) |
| LTU | MSE | 4178.627 (217.586) | 4106.44 (235.75) |
| LTU | RMSE | 64.612 (1.417) | 64.05 (1.81) |
| POL | R² | 0.462 (0.016) | 0.454 (0.028) |
| POL | MAE | 56.139 (1.159) | 57.25 (1.28) |
| POL | MSE | 4966.782 (201.297) | 5045.04 (226.81) |
| POL | RMSE | 70.457 (1.415) | 71.39 (1.59) |

*Note: Values are mean (SD) across 20 repeated school-grouped outer splits. The ten plausible-value-specific metrics were combined within each split. KNN denotes the primary imputation workflow. Median/mode denotes the full alternative-imputation workflow, in which continuous and categorical variables were imputed from the corresponding outer-training data. This table reports the held-out performance sensitivity to alternative imputation; the high-missingness quartile procedure was a separate screening-only stress test and is not represented here.*

**Supplementary Table S14. GA-BPNN SHAP sensitivity to alternative training-only imputation for the eight variables recurrent under the primary top-35 screening procedure.**

| **Education system** | **Variable** | **KNN mean \|SHAP\| (SD)** | **Median/mode mean \|SHAP\| (SD)** | **KNN mean rank (SD)** | **Median/mode mean rank (SD)** | **Rank difference (SD)** |
| --- | --- | --- | --- | --- | --- | --- |
| AUS | ESCS | 1.28 (0.69) | 1.43 (0.48) | 31.85 (4.36) | 30.95 (3.38) | -0.90 (6.47) |
| AUS | HOMEPOS | 3.37 (0.88) | 1.35 (0.17) | 19.25 (4.64) | 32.05 (1.43) | 12.80 (4.67) |
| AUS | MATHEFF | 7.33 (0.57) | 12.03 (0.53) | 3.20 (1.64) | 1.70 (0.47) | -1.50 (1.67) |
| AUS | ST255Q01JA | 5.89 (0.32) | 4.89 (0.27) | 7.95 (1.32) | 9.15 (0.81) | 1.20 (1.51) |
| AUS | ST290Q03WA | 3.17 (0.33) | 2.02 (0.16) | 21.00 (1.86) | 26.15 (1.39) | 5.15 (2.03) |
| AUS | ST290Q05WA | 3.81 (0.37) | 3.04 (0.29) | 16.70 (2.11) | 19.80 (2.38) | 3.10 (2.83) |
| AUS | ST290Q07WA | 1.22 (0.31) | 1.72 (0.28) | 32.50 (1.24) | 29.05 (2.56) | -3.45 (2.80) |
| AUS | ST290Q09WA | 3.93 (0.46) | 0.00 (0.00) | 15.55 (2.96) | 36.00 (0.00) | 20.45 (2.96) |
| BEL | ESCS | 1.66 (0.68) | 1.04 (0.37) | 29.35 (4.61) | 31.35 (2.56) | 2.00 (4.03) |
| BEL | HOMEPOS | 3.82 (1.00) | 3.13 (0.53) | 14.65 (5.76) | 14.25 (3.63) | -0.40 (5.83) |
| BEL | MATHEFF | 6.53 (0.57) | 6.22 (0.53) | 3.80 (1.11) | 5.10 (0.45) | 1.30 (1.13) |
| BEL | ST255Q01JA | 4.38 (0.35) | 2.88 (0.26) | 11.75 (1.68) | 16.25 (2.36) | 4.50 (1.67) |
| BEL | ST290Q03WA | 3.39 (0.40) | 0.33 (0.60) | 16.70 (1.89) | 34.25 (3.35) | 17.55 (3.59) |
| BEL | ST290Q05WA | 1.51 (0.48) | 1.45 (0.31) | 30.55 (2.78) | 28.40 (2.84) | -2.15 (3.94) |
| BEL | ST290Q07WA | 0.96 (0.24) | 0.00 (0.00) | 33.70 (1.38) | 36.00 (0.00) | 2.30 (1.38) |
| BEL | ST290Q09WA | 1.76 (0.53) | 0.00 (0.00) | 28.45 (3.52) | 36.00 (0.00) | 7.55 (3.52) |
| CZE | ESCS | 2.12 (0.68) | 2.10 (0.74) | 27.30 (4.89) | 24.55 (5.03) | -2.75 (7.89) |
| CZE | HOMEPOS | 4.98 (0.81) | 4.15 (0.48) | 8.95 (4.76) | 10.65 (2.30) | 1.70 (5.43) |
| CZE | MATHEFF | 5.26 (0.94) | 9.31 (0.54) | 7.80 (2.80) | 2.30 (0.47) | -5.50 (2.93) |
| CZE | ST255Q01JA | 5.52 (0.36) | 6.30 (0.45) | 6.15 (1.87) | 5.40 (0.68) | -0.75 (1.86) |
| CZE | ST290Q03WA | 3.78 (0.77) | 1.51 (0.15) | 15.55 (4.63) | 28.75 (1.52) | 13.20 (4.55) |
| CZE | ST290Q05WA | 1.62 (0.41) | 0.80 (0.10) | 31.20 (2.93) | 33.60 (1.05) | 2.40 (2.89) |
| CZE | ST290Q07WA | 1.30 (0.28) | 0.00 (0.00) | 32.70 (1.45) | 36.00 (0.00) | 3.30 (1.45) |
| CZE | ST290Q09WA | 2.56 (0.72) | 0.00 (0.00) | 23.65 (6.01) | 36.00 (0.00) | 12.35 (6.01) |
| FIN | ESCS | 1.46 (0.42) | 1.87 (0.82) | 31.20 (2.57) | 28.00 (4.48) | -3.20 (4.47) |
| FIN | HOMEPOS | 4.78 (0.73) | 2.36 (0.42) | 11.35 (3.17) | 25.05 (2.19) | 13.70 (3.89) |
| FIN | MATHEFF | 3.37 (0.99) | 8.05 (0.89) | 19.25 (5.84) | 3.90 (1.02) | -15.35 (5.72) |
| FIN | ST255Q01JA | 5.94 (0.36) | 4.87 (0.34) | 6.85 (1.39) | 11.45 (1.93) | 4.60 (2.09) |
| FIN | ST290Q03WA | 1.86 (0.30) | 1.75 (0.21) | 28.70 (2.34) | 28.40 (1.35) | -0.30 (2.49) |
| FIN | ST290Q05WA | 1.39 (0.36) | 1.28 (0.36) | 31.55 (2.24) | 31.10 (2.45) | -0.45 (3.03) |
| FIN | ST290Q07WA | 1.15 (0.28) | 1.06 (0.17) | 33.35 (1.53) | 32.70 (1.45) | -0.65 (1.81) |
| FIN | ST290Q09WA | 2.93 (0.48) | 0.00 (0.00) | 20.85 (3.33) | 36.00 (0.00) | 15.15 (3.33) |
| GBR | ESCS | 2.18 (1.19) | 1.82 (0.65) | 26.55 (8.26) | 26.90 (5.19) | 0.35 (10.00) |
| GBR | HOMEPOS | 2.35 (0.86) | 4.35 (0.57) | 25.65 (5.82) | 10.80 (2.35) | -14.85 (6.29) |
| GBR | MATHEFF | 6.48 (1.31) | 8.52 (1.12) | 5.75 (2.40) | 2.90 (0.79) | -2.85 (2.23) |
| GBR | ST255Q01JA | 5.36 (0.47) | 8.56 (0.80) | 7.50 (1.70) | 2.85 (0.93) | -4.65 (1.69) |
| GBR | ST290Q03WA | 2.96 (0.66) | 1.24 (0.13) | 20.30 (4.40) | 32.15 (1.35) | 11.85 (4.21) |
| GBR | ST290Q05WA | 1.76 (0.43) | 2.12 (0.40) | 29.45 (3.20) | 24.70 (3.33) | -4.75 (3.75) |
| GBR | ST290Q07WA | 1.63 (0.41) | 1.31 (0.21) | 30.75 (3.74) | 31.30 (2.00) | 0.55 (3.52) |
| GBR | ST290Q09WA | 3.74 (0.85) | 0.00 (0.00) | 15.70 (5.34) | 36.00 (0.00) | 20.30 (5.34) |
| IRL | ESCS | 1.84 (0.82) | 2.32 (0.69) | 26.35 (6.17) | 22.70 (5.09) | -3.65 (6.81) |
| IRL | HOMEPOS | 2.06 (0.34) | 1.68 (0.31) | 24.60 (2.60) | 27.85 (2.80) | 3.25 (3.60) |
| IRL | MATHEFF | 7.88 (0.88) | 9.59 (0.82) | 2.25 (1.02) | 1.05 (0.22) | -1.20 (1.01) |
| IRL | ST255Q01JA | 5.86 (0.48) | 5.09 (0.44) | 6.70 (1.92) | 8.75 (1.92) | 2.05 (1.39) |
| IRL | ST290Q03WA | 2.62 (0.32) | 2.12 (0.28) | 20.50 (2.26) | 23.45 (2.37) | 2.95 (2.44) |
| IRL | ST290Q05WA | 2.12 (0.29) | 1.92 (0.28) | 24.10 (3.08) | 25.30 (2.68) | 1.20 (3.38) |
| IRL | ST290Q07WA | 1.32 (0.23) | 1.55 (0.26) | 31.45 (2.37) | 28.75 (2.53) | -2.70 (3.11) |
| IRL | ST290Q09WA | 2.99 (0.48) | 2.58 (1.04) | 18.25 (2.94) | 20.45 (7.40) | 2.20 (8.16) |
| KOR | ESCS | 1.53 (0.41) | 2.79 (0.96) | 30.65 (4.31) | 21.80 (8.14) | -8.85 (9.21) |
| KOR | HOMEPOS | 5.12 (1.10) | 5.43 (1.50) | 9.25 (3.96) | 9.35 (5.26) | 0.10 (4.68) |
| KOR | MATHEFF | 10.48 (1.07) | 12.35 (1.01) | 2.20 (0.62) | 1.50 (0.51) | -0.70 (0.80) |
| KOR | ST255Q01JA | 4.05 (0.33) | 3.82 (0.49) | 13.25 (1.59) | 12.70 (2.72) | -0.55 (2.63) |
| KOR | ST290Q03WA | 2.95 (0.93) | 2.12 (0.51) | 18.90 (6.29) | 26.05 (5.03) | 7.15 (6.29) |
| KOR | ST290Q05WA | 1.49 (0.39) | 0.00 (0.00) | 31.50 (3.62) | 36.00 (0.00) | 4.50 (3.62) |
| KOR | ST290Q07WA | 1.56 (0.30) | 0.00 (0.00) | 30.40 (2.60) | 36.00 (0.00) | 5.60 (2.60) |
| KOR | ST290Q09WA | 3.04 (0.68) | 0.29 (0.90) | 18.65 (4.22) | 34.15 (5.71) | 15.50 (6.73) |
| LTU | ESCS | 3.00 (0.69) | 5.97 (0.59) | 19.10 (5.09) | 6.60 (2.50) | -12.50 (4.83) |
| LTU | HOMEPOS | 1.16 (0.33) | 3.56 (0.37) | 32.95 (1.82) | 17.45 (3.20) | -15.50 (3.69) |
| LTU | MATHEFF | 5.75 (0.70) | 5.36 (0.53) | 4.65 (2.41) | 8.50 (1.54) | 3.85 (2.64) |
| LTU | ST255Q01JA | 5.07 (0.31) | 4.51 (0.37) | 7.50 (1.19) | 11.55 (1.64) | 4.05 (1.50) |
| LTU | ST290Q03WA | 3.06 (0.38) | 1.44 (0.19) | 18.25 (3.49) | 30.90 (1.07) | 12.65 (3.10) |
| LTU | ST290Q05WA | 1.70 (0.32) | 1.17 (0.18) | 29.55 (2.48) | 32.65 (1.35) | 3.10 (2.67) |
| LTU | ST290Q07WA | 1.62 (0.28) | 1.58 (0.26) | 30.25 (2.27) | 30.40 (1.79) | 0.15 (2.41) |
| LTU | ST290Q09WA | 3.77 (0.35) | 3.22 (0.42) | 12.55 (2.19) | 20.45 (4.01) | 7.90 (4.17) |
| POL | ESCS | 2.70 (1.35) | 3.18 (0.95) | 21.45 (8.46) | 17.30 (4.93) | -4.15 (6.98) |
| POL | HOMEPOS | 2.14 (0.56) | 2.46 (0.53) | 23.60 (4.73) | 21.65 (2.46) | -1.95 (5.75) |
| POL | MATHEFF | 6.46 (0.67) | 5.17 (0.77) | 4.45 (1.76) | 8.85 (2.03) | 4.40 (1.57) |
| POL | ST255Q01JA | 5.83 (0.60) | 4.93 (0.52) | 6.30 (2.25) | 8.90 (1.52) | 2.60 (2.09) |
| POL | ST290Q03WA | 5.13 (0.60) | 2.90 (0.27) | 8.80 (2.21) | 18.50 (1.73) | 9.70 (2.62) |
| POL | ST290Q05WA | 1.63 (0.40) | 1.08 (0.32) | 28.40 (3.38) | 30.80 (3.16) | 2.40 (3.98) |
| POL | ST290Q07WA | 1.71 (0.47) | 0.05 (0.22) | 27.80 (4.49) | 35.80 (0.89) | 8.00 (4.59) |
| POL | ST290Q09WA | 2.52 (0.31) | 2.06 (0.30) | 20.60 (2.91) | 23.20 (2.21) | 2.60 (4.04) |

*Note: Values are mean (SD) across 20 repeated school-grouped outer splits after plausible-value-specific SHAP summaries were combined within each split. The same GA-BPNN SHAP protocol was applied separately under both imputation workflows: DeepExplainer; 256 outer-training background observations; all outer-test students; W_FSTUWT-weighted mean absolute SHAP aggregation; and signed one-hot contribution collapse to the original-variable level. The table reports all eight primary recurrent variables: ESCS, HOMEPOS, MATHEFF, ST255Q01JA, ST290Q03WA, ST290Q05WA, ST290Q07WA, and ST290Q09WA. The four sensitivity-supported variables are ESCS, HOMEPOS, MATHEFF, and ST255Q01JA; they do not replace the eight primary variables or imply invariance of exact ranks or attribution magnitudes. Lower rank denotes higher importance. Rank difference = median/mode − KNN. A rank of 36 denotes that the variable was not selected in that split under the relevant imputation workflow and was assigned zero SHAP importance; it is not an observed 36th rank.*

**Supplementary Table S15. Cross-architecture agreement between GA-BPNN SHAP and XGBoost TreeSHAP under the primary KNN workflow.**

| **Education system** | **Paired splits** | **Top-10 Jaccard overlap** | **Feature-rank Spearman correlation** |
| --- | --- | --- | --- |
| AUS | 20 | 0.697 (0.128) | 0.789 (0.064) |
| BEL | 20 | 0.774 (0.090) | 0.838 (0.033) |
| CZE | 20 | 0.693 (0.096) | 0.757 (0.037) |
| FIN | 20 | 0.609 (0.132) | 0.851 (0.039) |
| GBR | 20 | 0.721 (0.086) | 0.851 (0.052) |
| IRL | 20 | 0.841 (0.109) | 0.885 (0.020) |
| KOR | 20 | 0.753 (0.105) | 0.881 (0.029) |
| LTU | 20 | 0.702 (0.109) | 0.801 (0.038) |
| POL | 20 | 0.659 (0.106) | 0.759 (0.043) |
| Fixed cohort mean | 180 | 0.716 (0.123) | 0.824 (0.061) |

*Note: Values are mean (SD) across 20 paired school-grouped outer splits, except the final row, which summarizes all 180 paired splits across the fixed nine-system cohort. Both models used the primary KNN preprocessing workflow. Top-10 Jaccard overlap is the overlap of the 10 highest-ranked original variables. Feature-rank Spearman correlation compares ranks across the 35 primary screening variables within each paired split; it is not restricted to the eight primary recurrent variables or the four sensitivity-supported variables. Exact SHAP magnitudes and ranks remain model-conditional.*
